# The Pel Polysaccharide Can Serve a Structural and Protective Role in the Biofilm Matrix of *Pseudomonas aeruginosa*

**DOI:** 10.1371/journal.ppat.1001264

**Published:** 2011-01-27

**Authors:** Kelly M. Colvin, Vernita D. Gordon, Keiji Murakami, Bradley R. Borlee, Daniel J. Wozniak, Gerard C. L. Wong, Matthew R. Parsek

**Affiliations:** 1 Department of Microbiology, University of Washington, Seattle, Washington, United States of America; 2 Department of Physics, University of Texas, Austin, Austin, Texas, United States of America; 3 Department of Microbiology, Ohio State University, Columbus, Ohio, United States of America; 4 Department of Bioengineering, University of California, Los Angeles, Los Angeles, California, United States of America; Massachusetts General Hospital and Harvard Medical School, United States of America

## Abstract

Bacterial extracellular polysaccharides are a key constituent of the extracellular matrix material of biofilms. *Pseudomonas aeruginosa* is a model organism for biofilm studies and produces three extracellular polysaccharides that have been implicated in biofilm development, alginate, Psl and Pel. Significant work has been conducted on the roles of alginate and Psl in biofilm development, however we know little regarding Pel. In this study, we demonstrate that Pel can serve two functions in biofilms. Using a novel assay involving optical tweezers, we demonstrate that Pel is crucial for maintaining cell-to-cell interactions in a PA14 biofilm, serving as a primary structural scaffold for the community. Deletion of *pelB* resulted in a severe biofilm deficiency. Interestingly, this effect is strain-specific. Loss of Pel production in the laboratory strain PAO1 resulted in no difference in attachment or biofilm development; instead Psl proved to be the primary structural polysaccharide for biofilm maturity. Furthermore, we demonstrate that Pel plays a second role by enhancing resistance to aminoglycoside antibiotics. This protection occurs only in biofilm populations. We show that expression of the *pel* gene cluster and PelF protein levels are enhanced during biofilm growth compared to liquid cultures. Thus, we propose that Pel is capable of playing both a structural and a protective role in *P. aeruginosa* biofilms.

## Author Summary

Most bacteria live within biofilm communities, which are a complex population of microorganisms that attach to surfaces and produce copious amounts of extracellular matrix material. Exopolysaccharides are a key feature of the extracellular matrix and are found in many forms, ranging from structurally simple linear homopolymers to structurally complex branched heteropolymers. Exopolysaccharides carry out a wide range of functions involving adherence to surfaces and other cells, structural support and protection against host and environmental stress. The goal of our study was to examine the functional importance of polysaccharide production in the model biofilm organism, *Pseudomonas aeruginosa*. Using a deletion and over expression strategy, we characterized the function of one polysaccharide, Pel, and demonstrated that this polysaccharide has two roles, a structural role and a protective role, against an important class of antibiotics, aminioglycosides.

## Introduction

Biofilms are surface associated communities embedded within an extracellular matrix [1], [2], [3]. Biofilm communities exhibit enhanced antibiotic tolerance [4], [5], [6]. As a result, biofilm infections tend to be chronic and difficult to eradicate [2], [7]. This enhanced tolerance is thought to be multi-factorial, owing to biofilm-associated patterns of gene expression, slow growth rate, and reduced antimicrobial diffusion within the biofilm [4]. A focus of research has been to identify biofilm-associated factors that contribute to their antibiotic tolerance.

The opportunistic pathogen, *Pseudomonas aeruginosa*, is a model organism in biofilm research. *P. aeruginosa* is well known for the chronic infections it causes in individuals with the genetic disease, cystic fibrosis (CF) [7]. Biofilm formation within the CF airways is believed to facilitate the infection, helping the bacteria to withstand aggressive antimicrobial treatment and host defenses [8], [9].

The extracellular matrix is a distinguishing feature of biofilms, capable of functioning as both a structural scaffold and protective barrier to antimicrobials [1], [2], [10], [11], [12]. A key component of the matrix is extracellular polysaccharides [13]. Exopolysaccharides carry out a wide range of functions involving surface and cell-cell interactions, as well as protecting against antimicrobials and host defenses [3], [10], [14], [15], [16]. *P. aeruginosa* produces three exopolysaccharides, alginate, Pel and Psl, all of which have been implicated in biofilm development under different circumstances [11].

Pel's composition has yet to be fully elucidated. Initial carbohydrate analysis suggests Pel is a glucose-rich polysaccharide polymer although the exact structure remains unknown [17]. Pel synthesis machinery is encoded by a seven gene operon (*pelA-F*) originally identified in a mutagenesis screen for the loss of pellicle formation in PA14 [17]. Pel also appears to be important in static microtiter dish biofilm assays. A *pel* mutant strain had a defect in biofilm biomass accumulation in comparison to wild-type PA14 [17], [18]. The mechanism behind this observation remains unclear. Other studies have demonstrated that in the absence of type IV pili, Pel can play a role in attachment suggesting it can compensate as an attachment factor in the absence of other adhesins [18].

In this study, we conducted an analysis of Pel function. We focused our study on two common laboratory strains, PAO1 and PA14. PAO1 is capable of making both the Pel and Psl exopolysaccharides, while PA14 is only capable of producing Pel since three genes of the *psl* operon are deleted in this strain. We show that Pel is critical for maintaining cell-cell interactions in developing PA14 biofilms as well as providing protection against aminoglycoside antibiotics during biofilm growth. We also show that Pel does not appear to play any critical role in PAO1 biofilm development, where Psl appears to be the primary biofilm polysaccharide. Finally, we demonstrate that the *pel* operon is transcriptionally induced and PelF protein levels increase during biofilm growth. Thus, we propose that Pel can serve both as a structural and protective factor within a biofilm community.

## Results

### Generation of a *pel*-inducible overexpression strain

To initiate our study, we constructed a Pel overexpression strain. The native promoter region of *pelA* was replaced with the *araC*-P_BAD_ promoter on the chromosome in two common laboratory strains, PAO1 and PA14, allowing arabinose-dependent expression of the *pel* operon (**Figure S1A**). PA14 is a clinical strain obtained from a burn patient that has a multi-gene truncation in the N-terminal region of the *psl* operon and is incapable of synthesizing the Psl polysaccharide [19]. Accordingly, PA14 serves as a useful strain to study the contribution of the Pel polysaccharide independently of Psl. In contrast, PAO1 has the necessary genes to produce both polysaccharides. The inducible strains will be referred to as PAO1P_BAD_ *pel* and PA14P_BAD_ *pel*. Quantitative RT-PCR was used to quantify *pelA* transcript level from log phase cells. The level of *pelA* transcript increased with increasing concentrations of the inducer, arabinose (**Figure S1C**). The dose-dependent increase in transcription level was similar between PA14 and PAO1. Addition of 0.2% arabinose led to 51- and 61-fold increase in expression levels in PA14P_BAD_ *pel* and PAO1P_BAD_ *pel*, respectively. *pelA* transcript is expressed 1.8 times higher in wild-type PA14 compared to wild-type PAO1 relative to the internal control transcript, *ampR* (**Figure S1C**).

### Pel overexpression contributes to aggregation, Congo red binding, pellicle formation and rugose colony morphology

We evaluated PAO1P_BAD_ *pel* and PA14P_BAD_ *pel* for the ability to conditionally produce more Pel polysaccharide with increasing *pel* transcription. Previous work has demonstrated that Pel synthesis is controlled at multiple levels, transcriptionally and allosterically [20], [21]. Congo red binding and liquid culture aggregation are two phenotypes associated with increased polysaccharide production in multiple bacterial species [20], [22], [23]. Addition of 1% arabinose to both PA14P_BAD_ *pel* and PAO1P_BAD_ *pel* leads to bacterial aggregation in liquid culture relative to the uninduced strain and these bacterial aggregates hyperbind Congo red (**Figure S1B**).

Pel expression was previously demonstrated to impact pellicle formation and colony morphology [17]. Wild-type PA14 forms a distinct pellicle after about two days of incubation at room temperature which becomes more pronounced over time. When induced, PA14P_BAD_ *pel* rapidly produces a thicker pellicle compared to wild-type. A top-down view reveals that the PA14P_BAD_ *pel* stain produces a pellicle with a highly defined wrinkly architecture that is resistant to extensive vortexing (**Figure S1D, bottom panel**). Consistent with previously published data, a mutation in *pelB* leads to a dramatic reduction in pellicle formation compared to the parental strain [17], [24]. Pellicles produced in wild-type PAO1 grown under the same conditions are less distinct than PA14 pellicles. No discernable difference in pellicle formation is seen between PAO1 and PAO1Δ*pelB*, but overexpressing Pel enhances pellicle formation similar to PA14 (**Figure S1D**). Overexpression of *pel* produces enhanced wrinkly colony morphology in PA14, whereas the *pelB* mutant grows as a smooth colony with little Congo red binding (**Figure S1D, top panel**). Unexpectedly, PAO1 did not produce a wrinkly colony morphology for any of the tested strains despite many attempts with varying temperature and media conditions (**Figure S1D, top panel**).

### Pel contributes to biofilm development post-attachment

We investigated the function Pel plays in biofilm development using two biofilm culturing methods, a microtiter dish assay and a flow-cell reactor. A microtiter dish assay quantifies biofilm formation on plastic during static incubation. In contrast, a flow cell bioreactor allows a microscopic analysis of live biofilms growing in dilute medium under conditions of continuous flow.

**Figure 1:**
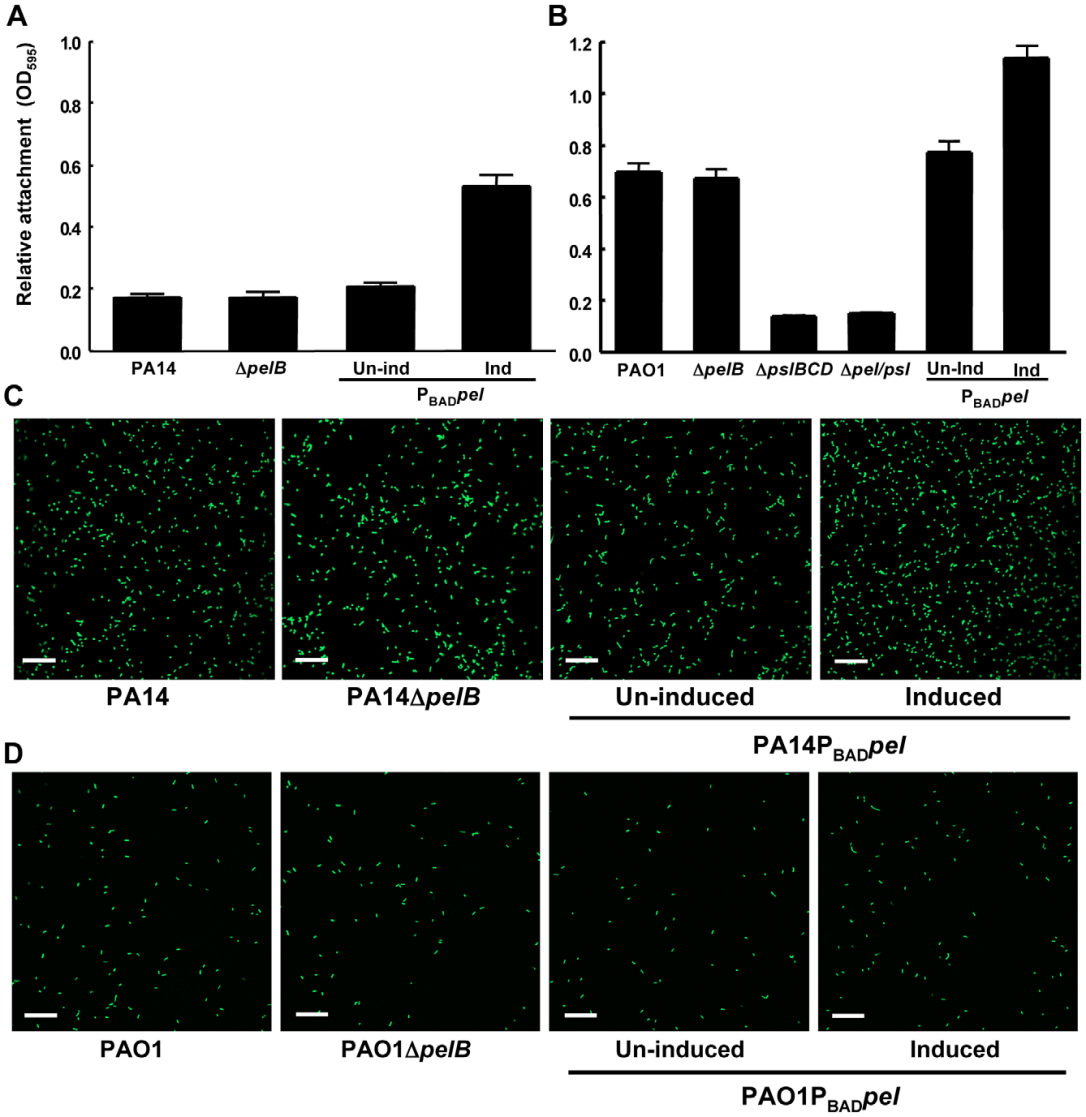
Pel is not required for attachment. Attachment of *P. aeruginosa* PA14 (A) and PAO1 (B) to microtiter dish wells was measured by crystal violet binding at an OD_595_. Overexpressing *pel* by addition of arabinose increased crystal violet binding in both PA14 and PAO1, but a *pelB* mutant showed no defect. Attachment to glass slides was visualized in a flow cell after one hour of attachment and one hour of continuous flow. Medium was supplemented with 0.2% arabinose under inducing conditions. Representative SCLM images of PA14, PA14Δ*pelB* and PA14P*_BAD_pel* are shown using a 40× objective (C). Representative SCLM images of PAO1, PAO1Δ*pelB* and PAO1P*_BAD_pel* are shown using a 40× objective (D). Scale bars represent 25 µm. Error bars represent standard deviations.

The influence of Pel on initial attachment to a plastic surface was examined. A *pelB* mutation in PA14 did not impact bacterial attachment (**Figure 1A**). However, overexpressing *pel* in either PA14 or PAO1 increased surface attachment (**Figure 1A and 1B**). Similar to PA14, no phenotype were seen in a PAO1Δ*pelB* mutant compared to wild-type PAO1 (**Figure 1B**). In contrast, but consistent with previously published work, a polar deletion in *psl* had a strong attachment defect in PAO1, indicating that Psl, and not Pel, is an important adhesin for surface attachment under these conditions [25], [26].

Crystal violet staining is an indirect measurement of bacterial attachment and thus we took a complementary, microscopic approach to evaluate Pel's role in attachment to a glass surface in a flow-cell reactor. Images were acquired by scanning confocal laser microscopy (SCLM) and analyzed by COMSTAT 1 software for surface coverage [27]. No statistical differences between PA14, PA14Δ*pelB*, uninduced and induced PA14P_BAD_ *pel* for attachment are observed (**Figure 1C** **and S2A**). Similar to PA14, no difference is observed for any of the PAO1 strains tested under non-inducing and inducing conditions **(** **Figure 1D** **and S2A)**. These results are slightly inconsistent with our microtiter dish assay, which demonstrate a modest but clear increase in attachment for the Pel overexpression strains. However, in both PAO1 and PA14, a *pel* mutation did not affect attachment in either biofilm culturing method.

**Figure 2:**
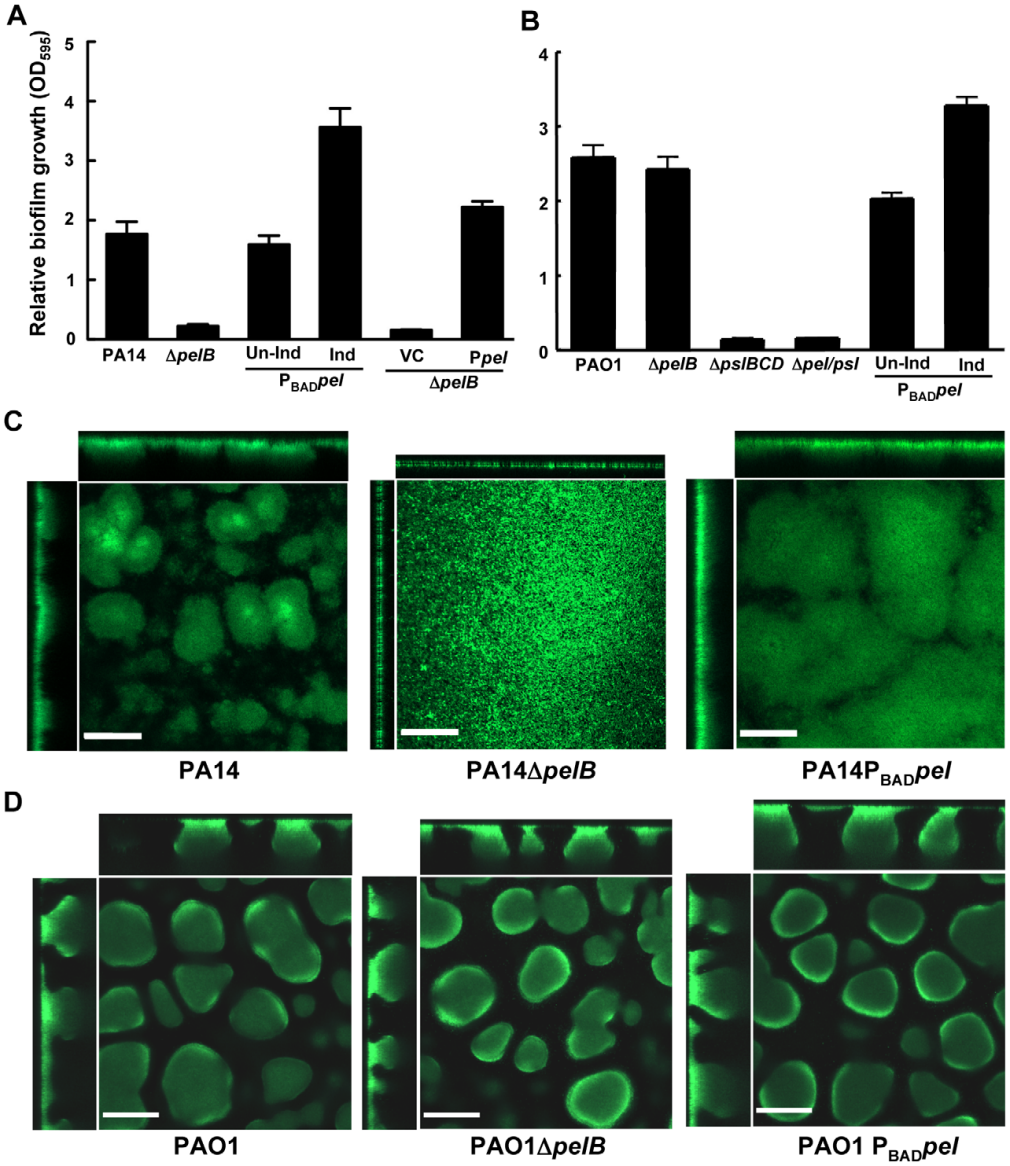
PA14Δ*pelB* is arrested in the monolayer stage of biofilm development. Strains PA14 (A) and PAO1 (B) were compared at 24 h for levels of biomass in microtiter dishes grown at room temperature by measuring crystal violet binding OD_595_. Expression of the *pel* operon from an arabinose-inducible plasmid pMJT1 (P*pel*) but not in the vector control (VC) alleviated the crystal violet staining defect of PA14Δ*pelB*. Biofilm structure was visualized in a flow cell and representative top-down and side-view images are shown for PA14, PA14Δ*pelB* and PA14P*_BAD_pel* (C) and PAO1, PAO1Δ*pelB* and PAO1P*_BAD_pel* (D). Images were obtained using a 20× objective after four d of growth in continuous flow chambers. Scale bars represent 100 µm. Error bars represent standard deviations.

Unlike surface attachment, we found that Pel has a significant impact on later stages of biofilm development and this impact was found to be strain dependent. To assess effects of Pel on later stages of biofilm development, we grew strains for 24 h in a microtiter dish assay and found that the *pelB* mutant strain of PA14 has a significant reduction in biofilm biomass compared to the parental PA14 strain, similar to previous findings (**Figure 2A**) [17], [18]. The PA14Δ*pelB* biofilm defect was complemented by supplying P*pel* in trans. P*pel* contains the entire *pel* operon cloned into an arabinose-controlled expression plasmid, pMJT-1 (**Figure 2A**). Overexpressing Pel in PA14 increased biofilm biomass almost two-fold (**Figure 2A**). In contrast to PA14, no difference is seen between PAO1 and PAO1Δ*pelB*, while overexpressing Pel results in a modest increase of biofilm biomass (**Figure 2B**). Conversely, PAO1Δ*pslBCD* has a pronounced defect, suggesting that Psl is the dominant polysaccharide in PAO1 for both attachment and biofilm maintenance, as reported by Ma, et al. [26].

Biofilm formation by these strains was monitored in a flow-cell bioreactor to allow for live imaging and structural analysis. Under these conditions, *P. aeruginosa* forms biofilms that contain mushroom-shaped multicellular structures. PA14 forms small microcolonies by day two that further develop into a structurally complex biofilm with large multicellular aggregates of bacteria by day four (**Figure 2C**). In stark contrast, PA14Δ*pelB* fails to form cellular aggregates. After four days of growth, PA14Δ*pelB* remains as a dense monolayer of cells attached to the glass surface, incapable of developing the complex three-dimensional structures typical of the wild-type strain (**Figure 2C**). The absence of cell aggregates in PA14Δ*pelB* indicates Pel may be responsible for the cell-to-cell adhesion necessary for aggregate formation. In support of this, overexpressing Pel results in larger cellular aggregates and enhanced biofilm biomass compared to wild-type PA14. Flow cell images were quantified for four properties of biofilm development using COMSTAT 1, average thickness, roughness coefficient, surface-to-volume ratio and maximum thickness (**Figure S2B**) [27]. Pel overexpression in PA14 affected each property by increasing the average thickness, decreasing the roughness coefficient, decreasing the surface to volume ratio and increasing the maximum thickness.

In contrast to PA14, no major visual or quantifiable difference is seen in biofilm structure after four days of growth for PAO1, PAO1Δ*pelB* and PAO1P_BAD_ *pel* (**Figures 2D** **and S2B**). However, a modest, but not statistically significant, increase in average biofilm thickness is detected for PAO1P_BAD_ *pel*. We subsequently assessed whether a Pel-dependent phenotype might manifest itself in older biofilms. Yet, even after nine days no significant differences were observed for PAO1, PAO1Δ*pelB* and PAO1P_BAD_ *pel* (**Figure S3**).

### Continuous expression of *pel* is required for continued biofilm growth, but not for maintenance of existing biofilm structure

Continuous production of the Psl polysaccharide was recently shown to be required for both the addition of new biofilm biomass to a growing biofilm and for the maintenance of existing biofilm structure [26]. Conditional loss of Psl expression resulted in a halt of biofilm growth and an eventual erosion of the existing biofilm structure [26].

**Figure 3:**
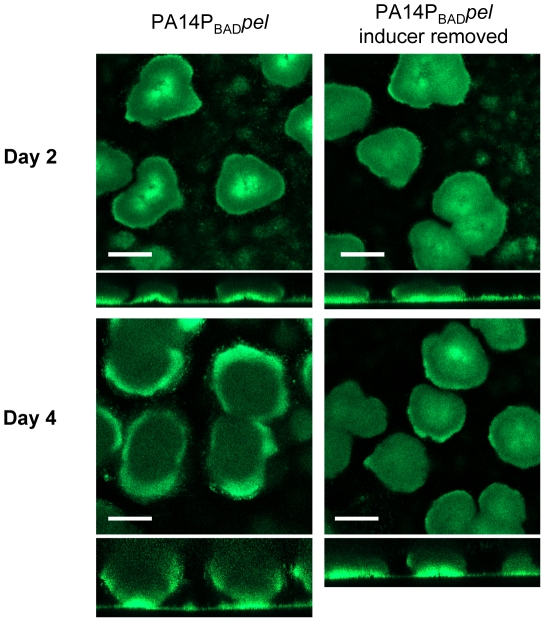
Continuous Pel production is required for biofilm growth, not maintenance of existing biofilm structure. PA14P_BAD_ *pel* was grown in 2% TSB for two days under inducing conditions (0.2% arabiniose). Biofilm growth was continued either in the presence (left) or absence (right) of the inducer arabinose. Representative top-down and side-view SCLM images from day two and day four are shown. Scale bars represent 100 µm.

Using our conditional expression system we grew PA14P_BAD_ *pel* biofilms for two days in the presence of arabinose and either continued providing arabinose to the biofilm culture for an additional two days or we removed it from the growth medium (**Figure 3**). Interestingly, halting Pel expression by removing arabinose resulted in a biofilm that failed to increase in size, but retained the original shape and mass after two days as calculated by COMSTAT (**Figure S4**). The biofilm that was supplied arabinose continued to grow in size. These results suggest that continuous Pel production is important over the course of biofilm development. However, unlike Psl, continuous Pel production is not required to maintain existing biofilm structure.

### Pel is critical for maintaining cell-to-cell interactions in PA14 biofilms

**Figure 4:**
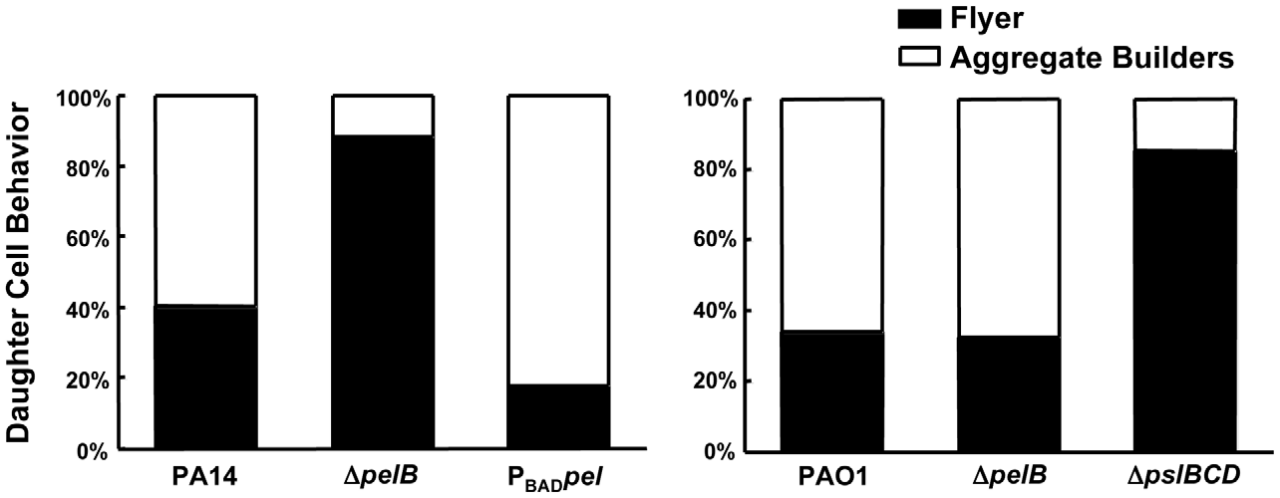
Pel impacts daughter cell behavior in early PA14 biofilms. Bacterial cell divisions were monitored by time-lapse microscopy in an early-stage biofilm grown in a flow cell. Daughter cells that remained within a 15 µm diameter of the mother cell are referred to as “aggregate builders”, other cells were termed “flyers”. A minimum of 75 cell divisions was assessed for each strain.

We hypothesized that the absence of cell aggregates in the PA14Δ*pelB* mutant biofilms is due to a defect in the cell-to-cell interactions necessary to hold an aggregate together. To initially test this hypothesis, we used time-lapse microscopy to analyze the behavior of biofilm cells at an early point in biofilm development. Dividing cells on the glass surface were monitored and the fate of daughter cells were separated into two categories [28]. Daughter cells that remained closely associated with the mother cell were termed “aggregate builders”. Cells that did not remain closely associated with the mother cells were designated “flyers”. We predicted if Pel were important in cell-to-cell interactions, cells incapable of Pel production would show a larger percentage of daughter cells exhibiting “flyer” behavior. Our analysis determined that Pel is a crucial determinant in daughter cell behavior in PA14 (**Figure 4**). As predicted, expression of Pel is related to daughter cell association with the parental cell. A *pelB* mutant displayed increased “flyer” behavior (88.3%) in comparison to wild-type PA14 (40.2%) and a reduced proportion of “aggregate builders” were observed (11.7%) than in PA14 (59.8%). Overexpressing *pel* resulted in an increased proportion of aggregate builders (82%) and relatively few flyers (18%).

Conversely, in PAO1 Psl appears to be the primary polysaccharide involved in aggregate building. PAO1 and the PAO1Δ*pel* mutant display indistinguishable daughter cell behavior profiles, with 33.9% and 32.6% of “flyers”, respectively. Conversely, PAO1Δ*pslBCD* exhibits a much larger proportion of “flyers” (85.3%) compared to PAO1 and PAO1Δ*pelB* (**Figure 4**). These data support our hypothesis that Pel contributes to aggregate formation in a PA14 biofilm by promoting retention of daughter cells within a growing aggregate, while Psl appears to be the critical polysaccharide for aggregate building in PAO1.

To complement the time-lapse microscopy study, we developed a novel assay involving infra-red laser [29]. This assay involves maintaining an optical trap in a liquid suspension of bacteria. Once bacteria enter the trap, they remain there. Initial experiments determined that continuous trapping of PA14 cells in liquid culture promoted the formation of stable aggregates. Using this technique we are able to study the effects of the Pel polysaccharide for the ability to form and maintain bacterial clusters. Since Pel was required for maintaining cell-to-cell interactions in flow cell biofilms, we predicted that Pel would be required for maintaining stable aggregates in this assay.

**Figure 5:**
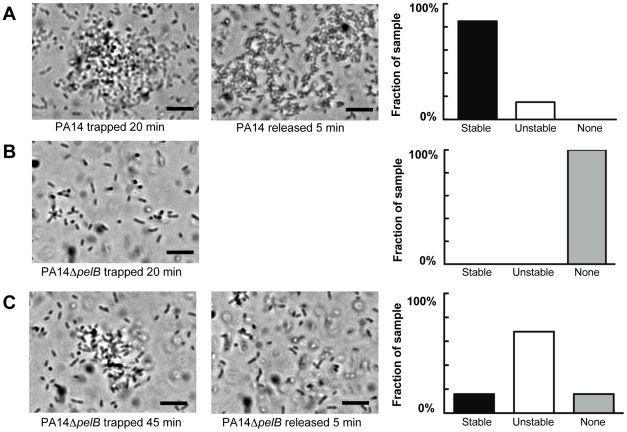
Pel is important for cell-to-cell interactions necessary for aggregate formation. Laser tweezers were used to trap bacteria and investigate bacterial clumping phenotypes. The captured bacteria were examined visually by light microscopy for aggregation after 20 min (A) PA14 (B) PA14Δ*pelB*. An extended trapping time of 45 min was required to initiate aggregation in PA14Δ*pelB* (C). The stability of formed aggregates was visually assessed five min after the release of the laser trap (center panel A and C). Aggregate stability was classified into three categories, “stable” if the aggregate remained intact, “unstable” if the aggregate dispersed into single cells and “none” if an aggregate did not form during the allotted time (right panels). A minimum of six replicates for each strain was assessed. Scale bars represent 10 µm. Representative phase-contrast images are shown.

Wild-type PA14 forms aggregates after 20 min of trapping in all visualized fields (**Figure 5A**). In contrast, PA14Δ*pelB* did not form aggregates, even though a significant amount of free-floating bacteria entered and remained in the trap (**Figure 5B**). Rather, the mutant strain requires a minimum of 45 min of trapping to form aggregates (**Figure 5C**). Even with the extended incubation in the trap, 16% of the fields of view are absent of aggregates. Based on the differences in time for bacterial clustering to be observed, these data conclude Pel is an important component in the initiation of cellular clustering. Subsequently, we tested to see if Pel was important in maintaining clustering aggregates after an aggregate was formed. These experiments were set up similarly by allowing bacterial aggregation to occur for 20 min for wild-type PA14 and 45 min for the *pelB* mutant. After the designated incubation time with the laser, cluster stability was monitored by microscopy five minutes after the laser trap was disengaged. More than six aggregates in each strain were visually assessed for stability and separated into three categories as described in the figure legend (**Figure 5**). 85% of wild-type PA14 cell aggregates are stable five min after the release of the trap. In contrast, only 16% of the PA14Δ*pelB* aggregates remained after the laser trap is removed. These results further support that Pel is critical for both initiating and maintaining cell-to-cell interactions.

### Pel provides biofilms protection from aminoglycoside antibiotics

A primary function attributed to the extracellular matrix is protection [12]. Several well-studied polysaccharides are known to confer resistance to a range of antibiotics. In *P. aeruginosa*, alginate and cyclic glucans have been demonstrated to protect biofilms from aminoglycosides by directly binding these cationic antibiotics [15], [30], [31]. In addition, rugose small colony variants (RSCVs), which produce elevated levels of Pel and Psl, show increased tolerance to tobramycin, an aminoglycoside [23], [32]. Thus, we hypothesized that Pel may provide protection from antimicrobials. Therefore we tested the sensitivity of our strains to several clinically relevant antibiotics: tobramycin, gentamicin, ciprofloxacin, kanamycin, meropenem, ceftazidime, tetracycline, and carbenicillin.

Planktonic cultures of PAO1, PAO1Δ*pelB*, PAO1Δ*psl*, PAO1P_BAD_ *pel* and WFPA801 (arabinose-inducible *psl* strain [26]) were initially tested for antibiotic susceptibility by determining the minimum inhibitory concentration (MIC) of each strain. No difference is detected between PAO1, PAO1Δ*pelB*, PAO1Δ*psl* and WFPA801 for any of the antibiotics tested (**Figure S5**). However, overexpressing Pel in PAO1 slightly increases the MIC in comparison to wild-type PAO1 to gentamicin and tobramycin, two aminoglycoside antibiotics. No difference is seen in MICs between PA14, PA14Δ*pelB* and PA14P_BAD_ *pel* for any of the antibiotics tested (**Figure S5**).

We then assessed Pel's involvement in planktonic survival by treating log-phase cultures of our strains with both tobramycin and ciprofloxacin. Ciprofloxacin was chosen as a representative antibiotic that has the same MIC for all three strains PAO1, PAO1Δ*pelB* and PAO1P_BAD_ *pel*. Equal susceptibility is seen between the wild-type and Δ*pelB* mutants in both PAO1 and PA14 (**Figure S6**). Like the MIC experiments, overexpression of Pel in PAO1 affords a small degree of protection to killing by tobramycin and gentamycin, while Pel overexpression in PA14 does not (**Figure S6**). Similar killing curves are observed between PA14 and PAO1 strains during ciprofloxacin treatment (**Figure S6**).

**Figure 6:**
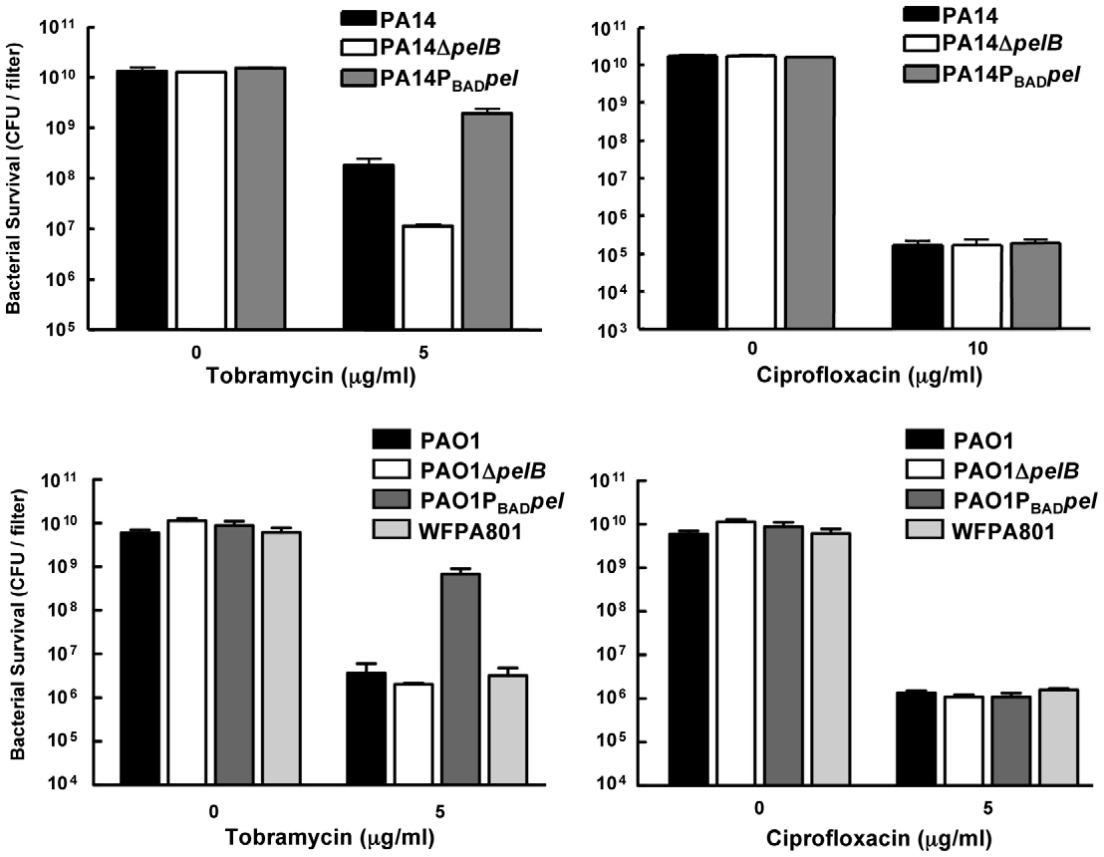
Analysis of Pel-mediated antibiotic tolerance in biofilms. 48-h filter biofilms were assessed for relative susceptibility. Biofilms were treated with tobramycin and ciprofloxacin for 24 h. No antibiotic controls are included for baseline comparison. WFPA801 over-expresses the Psl polysaccharide. Bacterial survival was measured by CFU counts.

We subsequently assessed Pel's role in antibiotic resistance in a biofilm model. For a valid comparison of antibiotic tolerance, the same number of cells must be challenged with the antibiotic of interest. In order to satisfy this criterion, we used a 48-h colony biofilm technique that has been demonstrated previously to capture a biofilm-specific model of antibiotic susceptibility [33]. Bacterial strains were grown on polycarbonate filters for two days, allowing complete coverage of the filter and equal colony forming units (CFUs) for all strains. The filter was then transferred to solid medium containing antibiotic and incubated for 24 hours. After treatment, the viable CFUs were quantified. In PA14, a *pel* mutation rendered biofilms more susceptible to the aminoglycosides tobramycin and gentamicin, while not impacting the susceptibility to ciprofloxacin **(** **Figures 6** **and S7)**. However, a *pel* mutation in PAO1 did not influence susceptibility to any antimicrobial tested.

Overexpressing *pel* in both PAO1 and PA14 led to an elevated tolerance to tobramycin and gentamicin compared to the corresponding parental strain (**Figures 6** **and S7**). To test whether Psl overexpression might provide similar aminoglycoside protection to PAO1, we used an arabinose-inducible Psl expression strain, WFPA801. Psl overexpression strain was found not to confer protection from tobramycin (**Figure 6**).

**Figure 7:**
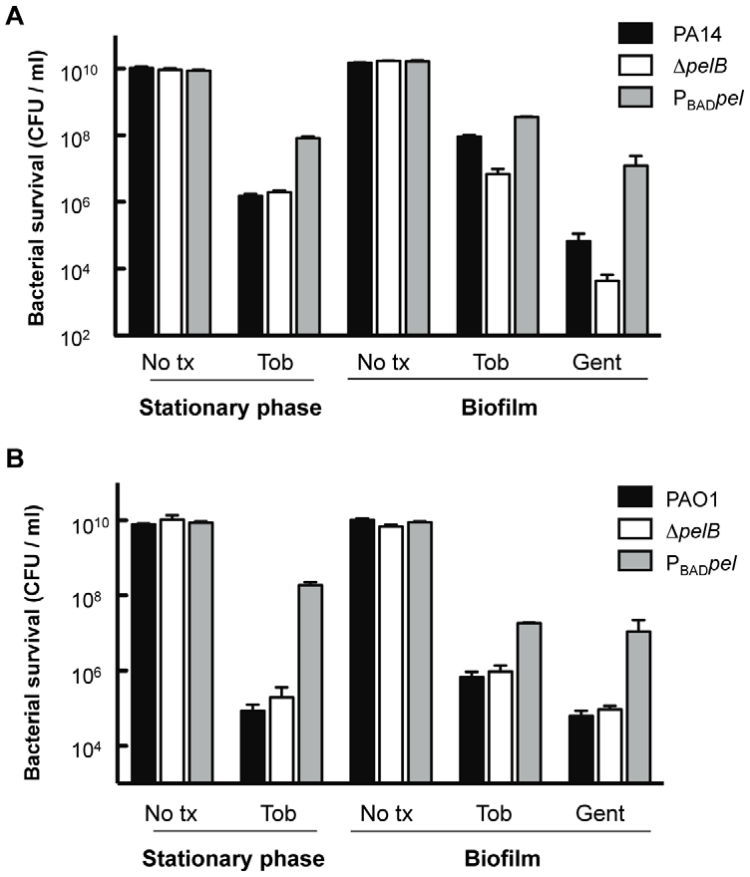
Analysis of Pel-mediated antibiotic resistance to stationary phase planktonic and biofilm grown cells. Bacterial survival was assessed for both PA14 (A) and PAO1 (B) 24 h stationary phase cultures and 24 h filter biofilms. Prior to antibiotic treatment, stationary phase planktonic cells were centrifuged and resuspended in fresh media containing no treatment (No tx) or indicated antibiotics. Biofilm cells were moved to a fresh media source containing no treatment (No tx) or antibiotics. Planktonic cultures were treated with 5 µg/ml tobramycin (Tob). Biofilm cells were treated with either 10 µg/ml of gentamicin (Gent) or tobramycin at 5 µg/ml for PAO1 and 150 µg/ml for PA14.

To make a more direct comparison between planktonic and biofilm cultures, we compared 24-h-old stationary phase cells with 24-h-old biofilm cells for tobramycin sensitivity. The tobramycin sensitivity profiles of stationary phase liquid cultures are identical for PAO1 and PA14 wild-type and the corresponding *pelB*-mutant strains **(** **Figure 7** **)**. Interestingly, overexpression of Pel in PA14 provides protection in stationary phase cells that is not observed in log phase cells (compare **Figures 7** **and S6**). Similar to log-phase treated cells, PAO1P_BAD_ *pel* provides protection to stationary phase cells. 24-h-biofilms reveal the same susceptibility profiles as the 48-h-treated biofilms shown in Figure 6, with enhanced sensitivity of PA14Δ*pelB* compared to wild-type (**Figure 7**).

To complement our analysis of colony biofilms, we determined the spatial distribution of tobramycin killing in flow cell biofilms. As expected, PA14 and its derivatives display a similar tobramycin resistance pattern as the filter biofilm. The *pelB* mutant strain produced a monolayer that is easily killed, while the PA14P_BAD_ *pel* strain biofilm is the least susceptible, probably in part due to the production of greater amounts of biofilm biomass than PA14 **(Figure S8)**.

### Expression of the *pel* operon is induced during biofilm growth

**Figure 8:**
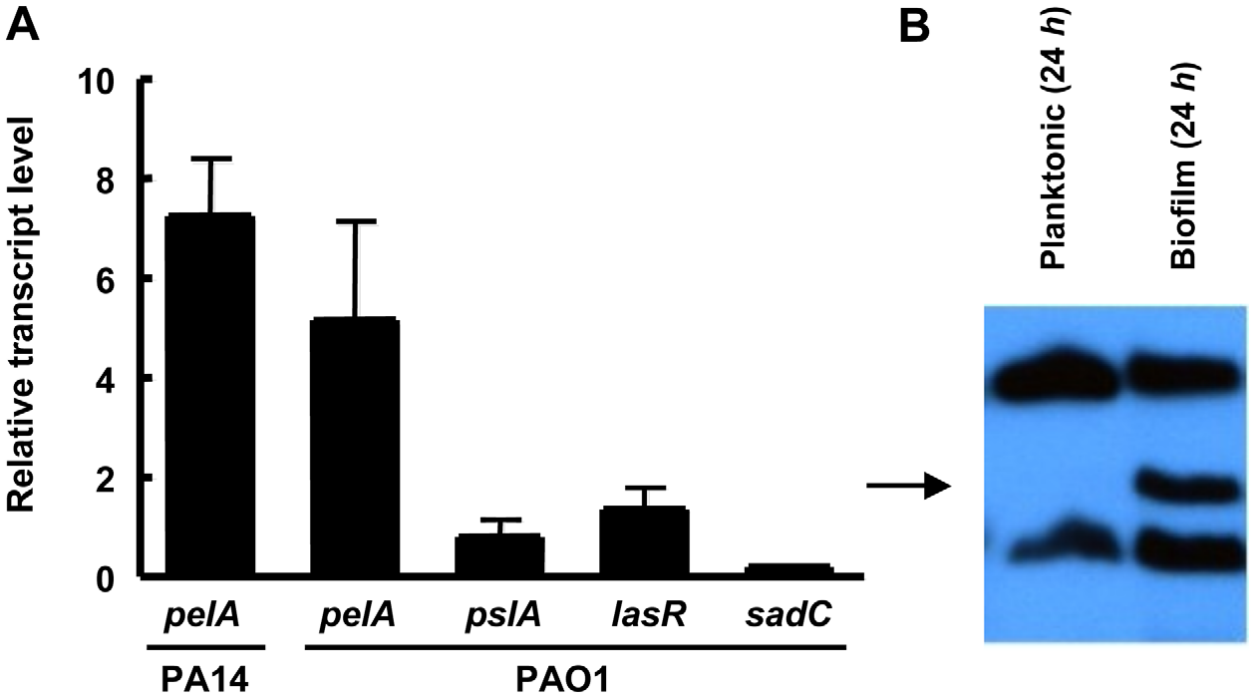
*pel* expression is elevated during biofilm growth. (A) Planktonic and biofilm cells are compared for transcript level by quantitative RT-PCR. In both conditions, bacteria are grown to log phase at 37°C. Planktonic cells are incubated statically in a test tube at room temperature for 30 min. Biofilm cells are grown in a tube biofilm, with an initial attachment period of 30 min followed by continuous flow for 48 h. Transcripts are normalized to *ampR* and then to the initial planktonic condition. Results shown are the mean of three independent experiments. Error bars represent the standard deviations. (B) Planktonic and biofilm cells were probed for PelF protein expression by western blot. A 24 h shaking liquid culture was compared to a 24 h grown tube biofilm at RT. Samples are normalized to total protein. The arrow indicates PelF expected protein size, 56 kDa.

Our antimicrobial tolerance data suggest that in PA14, Pel plays a more important role in biofilm communities as compared to planktonic cultures. One explanation for this observation is that *pel* expression may be enhanced during biofilm growth as compared to planktonic growth. To test this, we analyzed the expression of the *pel* operon in planktonic and biofilm cells using quantitative RT-PCR. To generate enough biofilm biomass for RT-PCR we grew the strains on the surface of silicon tubing under constant flow. We observed that *pelA* transcript in PA14 is 7.2-fold higher (+/−2.0) when grown as a biofilm for 48 h than in planktonic conditions (for either logarithmic or stationary phase cells), while in PAO1 it is 5.11-fold higher (+/−3.49). The control transcripts *pslA*, *lasR* and *sadC* did not exhibit biofilm-specific induction (**Figure 8A**). The *pslA* transcript was chosen as a control because the Psl polysaccharide is an important structural component in biofilm development in PAO1. The *lasR* transcript was chosen because LasR responds to an increase in biomass and the *sadC* transcript was chosen because the product SadC is a diguanylate cyclase important in regulating biofilm advancement [34], [35]. Therefore, an increase in *pelA* transcript is specific to the *pel* operon and not all genes involved in biofilm formation. Even by 24 h of biofilm growth, PAO1 shows nearly a 15-fold increase in *pelA* transcript **(Figure S9)**. To corroborate our transcriptional analysis, we also demonstrate PelF protein levels are elevated during biofilm growth but went undetected in stationary phase liquid cultures (**Figure 8B**). These data suggest a biofilm-associated role for Pel.

## Discussion

In this study, we have identified two key biofilm-associated functions of the Pel polysaccharide. Pel is critical for initiating and maintaining cell-cell interactions. These functions have been implicated in polysaccharides in other species, such as the MDX polysaccharide of *Shewanella oneidensis* and colanic acid of *E. coli* K-12 [36], [37]. This appears to be a crucial mechanism by which parent cells retain their daughter cells in the biofilm community. In the absence of Pel, biofilm formation does not progress beyond the monolayer stage in PA14.

In addition, Pel appears to provide a measure of protection from aminoglycoside antibiotics. The antibiotic susceptibility experiments suggest that Pel is capable of providing protection to planktonic cells when artificially overexpressed, although there is no phenotype for the *pel* mutant strain in liquid culture for any of the tested conditions. However, in biofilms of PA14, both *pel* overexpression and a *pel* mutation impacted aminoglycoside sensitivities. This suggests that Pel may play an important protective role in biofilms of this strain.

The mechanism responsible for protection is not clear, but if Pel behaves similarly to other polysaccharides leading to elevated aminoglycoside resistance like alginate and *ndvB*-encoded glucans, it may bind or sequester the antibiotic. Both alginate and *ndvB*-encoded glucans have a high negative charge that is consistent with their ability to bind positively charged aminoglycosides. If this model proves to be true, Pel may be an acidic polysaccharide capable of interacting with cationic antibiotics. This hypothesis helps explain why no differences were seen in killing of planktonic or biofilm cells by ciprofloxacin, an anionic antibiotic, and why no protection was afforded by overexpressing the neutral polysaccharide, Psl (**Figure 6**). Another possibility is that Pel production can influence biofilm structure, which in turn may influence antimicrobial susceptibility. However, we feel this is unlikely since the structure of colony biofilms tends to be uniform.

Using PAO1 and PA14 as representative *P. aeruginosa* laboratory strains, we see that the role Pel plays in biofilm formation can vary drastically. In PAO1, it appears that Psl is the predominate polysaccharide of the biofilm EPS matrix, while in PA14 Pel is required. However, it appears in other strains, MJK8, PAO1Δ*wspF* and ZK2870, both polysaccharides contribute to biofilm and/or autoaggregation phenotypes [20], [24], [32]. Whether each polysaccharide has a distinct role in biofilm formation and/or protection, or if their functions are redundant remain to be determined. Although purely in terms of surface attachment, it appears that Psl is more important, while Pel is less so.

The enhanced expression of Pel in biofilms is noteworthy. *pelA* transcript levels were minimally expressed under the planktonic culturing conditions we used. Yet despite low *pelA* transcript in planktonic conditions, only in biofilms do we detect PelF protein expression (**Figure 8**). Therefore, the protection afforded to *P. aeruginosa* by Pel from aminoglycosides appears to be a biofilm-associated mechanism of antimicrobial tolerance. To date, only the cyclic glucans encoded by the *ndvB* locus has been shown to be a biofilm-specific mechanism of antimicrobial tolerance in this species [30]. Characterizing the structure of Pel and the specific mechanism behind aminoglycoside protection is underway.

Finally, the ability to prohibit PA14 biofilms from growing larger by arresting Pel expression is exciting. The biofilm does not dissipate indicating that continuous Pel is not necessary for biofilm maintenance. This result is contrary to PAO1 biofilms that require Psl to be continuously produced for biofilm maintenance [26]. Thus, manipulating Pel and Psl expression may be a central strategy for disrupting biofilms and targeting them for antibiotic therapy.

## Materials and Methods

### Bacterial strains, strain construction, and growth media

Strains and primers used in this study are listed in **Table S1** [38], [39], [40], [41], [42], [43]. Plasmid and strain construction are described in **Text S1**. Unless otherwise noted, strains were grown at 37°C in LB medium. For plasmid selection, 300 µg/ml carbenicillin or 100 µg/ml gentamicin was used with *P. aeruginosa*, and 100 µg/ml ampicillin or 10 µg/ml gentamicin was used with *Escherichia coli*.

### RNA purification and analysis

RNA was extracted using an RNeasy kit (QIAGEN) according to manufactures instructions. Contaminating DNA was removed with an on-column RNase-free DNase I treatment (QIAGEN) and remaining DNA was removed by an off-column DNase I treatment (Promega) as recommended. The RNA prep was confirmed to be free of DNA by PCR. cDNA was generated by SuperScript III First-Strand Synthesis System for RT-PCR using random hexamers (Invitrogen). cDNA synthesis was verified by PCR and quantitated by RT-PCR using the SYBR Green PCR Master Mix (Applied Biosystems) as the fluorescent dye. Fluorescence was measured using ABI Prism 7000 Sequence Detection and *pelA* transcript levels were normalized to *ampR*.

### Microtiter dish biofilm

96-well microtiter dish experiments were performed as described previously [44]. For rapid attachment assays, 100 µl of log-phase cells were incubated at 37°C for one hour. For analysis of biofilm development, log-phase cells were incubated at room temperature for 20 h.

### Pellicle formation assay

Standing cultures containing 3-ml LB broth were grown at room temperature in a glass tube. Pellicles were monitored by visual inspection between four and ten d. Complete coverage at the air-liquid interface of an opaque layer of cells is considered to be indicative of pellicle formation [17].

### Congo red assays

LB liquid cultures supplemented with 40 µg/ml Congo red (Sigma-Aldrich) and incubated shaking overnight 37°C. The supernatants were measured at OD_495_ to assess Congo red binding. Congo red plates contained LB without NaCl, 1% agar, 40 µg/ml Congo red and 15 µg/ml brilliant blue R (Sigma-Aldrich). Cells were diluted 1/100, 10 µl spotted and incubated at room temperature for five d.

### Flow cell and tube biofilm reactor

The flow cell system and tube biofilm system was assembled as described previously [32], [45], [46]. Additional information is found in **Text S1**.

### Live/dead staining

Biofilms were grown in flow cells in 1% TSB as described in **Text S1** for 4 d and subsequently treated with 1 µg/ml of tobramycin for 24 h. The MIC for tobramycin when the cells are grown in 1% TSB is 0.03 µg/ml for PAO1 and 0.06 µg/ml for PA14. This is in contrast to the MIC of 1 µg/ml seen for PA14 and PAO1 grown in full strength LB. After treatment, flow was stopped and biofilms were stained with 500 µl of propidium iodide and SYTO 9 (Invitrogen) for 10 m according to manufactures instructions. Flow resumed and images were captured after 15 m of washing.

### Biofilm daughter-cell analysis

The movement and behavior of individual daughter cells of *P. aeruginosa* monocultures were monitored in young (<2 days old) flow cell biofilms. The flow cell setup was the same as described. Fluorescent images were taken every 45 seconds for 2 hours using the time-lapse feature of the Zeiss Axiophot microscope (Carl Zeiss). The fate of new daughter cells were visually tracked and each cell was classified as an “aggregate builder” or “flyer” [28]. From one cell division to the next, “aggregate builders” remained within a 15 µm diameter circle centered at the point of cell division, while “flyers” moved further away on the surface or were dissociated from the surface by media flow. A minimum of 75 cell divisions of each strain were tracked and classified.

### Laser trap experiments

An open-top chamber for microscopy was constructed by layering five Secure-Seal Imaging Spacers 13mm in diameter (Grace Bio-Labs) onto a microscope cover slip and this chamber placed on an inverted Olympus microscope above a 40× or 60× long-working-distance objective. Bacteria were grown shaking at 37°C to OD_600_ 1. Samples were incubated statically at room temperature for 1–7 hours. Static incubation of the cells prior to the experiment allowed for a slight increase (∼2-fold, data not shown) in *pel* expression, presumably for the same reasons *pel* expression is required for pellicle formation in static liquid cultures [17]. A 100 µL bacterial suspension was placed into the chamber. Laser trapping was done by focusing a 1064 nm laser through the microscope objective at the top of the sample at a transmitted power of ∼50mW. For PA14 wild-type bacteria, the first trap-induced clustering was seen in samples that had been in the open-top chamber for 20 min; for Δ*pelB* bacteria the first trap-induced clustering, if any, was seen in samples that had been in the open-top chamber for 45 min. Cluster stability was evaluated by monitoring formed bacterial aggregates for cluster dispersal five min after the laser beam was blocked.

### Immunoblot analysis

Immunoblots were performed with whole-cell lysates as described with equal amounts of total protein in each lane [47]. Protein concentration was measured using the Pierce 660 nm protein assay (Thermo Scientific). Lysates were probed for PelF expression levels with a specific PelF antibody. Additional information on antisera production and immunoblot analysis are described in **Text S1**.

### Antibiotic sensitivity assays

MIC growth curves were completed in a 96-well microtiter dish grown in LB broth at 37°C. Log phase bacteria were diluted to 10^5^ CFU in each well. A range of concentrations was assessed for each antibiotic. Bacterial growth was measured after 24 hours of incubation using a microplate reader (OD_590_).

For planktonic killing curves, cells were either grown to log phase in LB or grown for 24 h to assess stationary phase susceptibility. Log phase cultures were split and one culture was treated with either tobramycin (Sigma), gentamicin (Sigma) or ciprofloxacin (Bayer Healthcare), while the control culture was untreated. Stationary phase cells were resuspending in fresh media containing antibiotics. The cultures were incubated shaking at 37°C. Bacterial survival was assessed over time by viable plate counts.

For biofilm killing, overnight liquid cultures were diluted 1/100 and five µl were spotted onto a UV-sterilized 25mm polycarbonate filter (GE Osmonics). Biofilms were grown for two d unless otherwise stated at 37°C and moved to fresh solid media each day. Biofilms were exposed to with 5 µg/ml tobramycin or 1 µg/ml ciprofloxacin for 24 h. Bacterial viability was obtained by resuspending the filter in 1 ml of PBS and serially diluting to obtain viability counts.

## Supporting Information

Figure S1 Generation of a *pel*-conditional strain. (A) Schematic drawing showing the replacement of the promoter region with an arabinose promoter on the chromosome to produce PAO1P_BAD_ *pel*. (B) Overnight growth in the presence of 1% arabinose to PA14P_BAD_ *pel* and PAO1P_BAD_ *pel* leads to visual aggregates in liquid culture (right tubes) but not seen in the uninduced culture for either PA14P_BAD_ *pel* and PAO1P_BAD_ *pel* (left tubes). Congo red was added to visually enhance the phenotype. (C) Quantitative RT-PCR analysis of *pelA* transcription in PA14 and PA14P_BAD_ *pel* (left) and PAO1 and PAO1P_BAD_ *pel*(right) in the presence of increasing concentrations of arabinose. *pelA* transcription is normalized to *ampR* transcription. Results shown are the mean of three independent experiments. Error bars represent the standard deviations. (D) Colony morphology (top) and pellicle formation (middle, side-view; bottom, top-down) of PA14, PA14Δ*pelB* and PA14P_BAD_ *pel* (left) and PAO1, PAO1Δ*pelB* and PAO1P_BAD_ *pel* (right) grown in LB without NaCl containing 0.5% arabinose. Photographs were taken after five d of growth at room temperature.
(0.42 MB TIF)

Figure S2 Attachment and biofilm structure quantified by COMSTAT 1. (A) Using the substratum coverage variable in COMSTAT 1, the relative number of cells attached to the glass slide of a flow cell after one hour of attachment followed by one hour of continuous flow was measured. Four images per flow cell done in duplicate in three independent experiments were evaluated. (B) COMSTAT 1 assessed four variables of biofilm structure for PA14, PA14Δ*pelB*, PA14P_BAD_ *pel*, PAO1, PAO1Δ*pelB* and PAO1P_BAD_ *pel* for the SCLM image stacks of day four biofilms. Three images per flow cell done in duplicate in three independent experiments were evaluated.
(0.18 MB TIF)

Figure S3 Biofilm structure in nine-day PAO1 biofilms. (A) Biofilm structure was visualized by SCLM in a flow cell after 9 days of growth. Representative top-down and side-view images are shown for PAO1, PAO1Δ*pelB* and PAO1P*_BAD_pel*. Images were obtained using a 20× objective. Scale bars represent 100 µm. (B) Images were quantified for average thickness, roughness coefficient, surface to volume ratio and maximum thickness by COMSTAT 1. Four image stacks per flow cell done in duplicate in two independent experiments were evaluated.
(0.97 MB TIF)

Figure S4 COMSTAT 1 analysis of arabinose removal in PA14P_BAD_ *pel* flow cell biofilms. PA14P_BAD_ *pel* biofilms were grown for two days under inducing conditions (0.2% arabinose). Biofilms were either continued to be grown in the presence or absence of the inducer, arabinose. COMSTAT 1 evaluated SCLM images for average thickness, roughness coefficient, surface to volume ratio and maximum thickness. Four image stacks per flow cell done in duplicate in two independent experiments were evaluated.
(0.11 MB TIF)

Figure S5 Effect of Pel on the minimum inhibitory concentration (MICs) to a wide range of antimicrobials. Strains were assessed for their MIC by broth dilution to carbenicillin (Carb), ciprofloxacin (Cip), tobramycin (Tob), gentamicin (Gent), tetracycline (Tet), meropenem (Mero), kanamycin (Kan) and ceftazidime (Ceft). Concentrations shown are in µg/ml and were empirically determined. Bacterial strains were grown in the presence of 0.5% arabinose.
(0.11 MB TIF)

Figure S6 Analysis of Pel-mediated antibiotic tolerance in planktonic culture. Log-phase planktonic cultures were treated with either tobramycin (A), gentamicin (B) or ciprofloxacin (C). Bacterial survival was monitored over time by assessing the number of CFUs. On the left, PA14 (solid line), PA14Δ*pelB* (dashed line) and PA14P_BAD_ *pel* (dotted line) were treated with 5 µg/ml tobramycin, 2 µg/ml gentamicin and 0.1 µg/ml ciprofloxacin. On the right, PAO1 (solid line), PAO1Δ*pelB* (dashed line) and PAO1P_BAD_ *pel* (dotted line) were treated with 5 µg/ml tobramycin, 5 µg/ml gentamicin and 1 µg/ml ciprofloxacin.
(0.33 MB TIF)

Figure S7 Pel provides tolerance to gentamicin during biofilm growth. 48 h colony biofilms for PA14, PA14Δ*pelB* and PA14P*_BAD_pel* (A) and PAO1, PAO1Δ*pelB* and PAO1P*_BAD_pel* (B) were assessed for antibiotic susceptibility. Biofilms were treated with gentamicin for 24 h. No antibiotic addition is included for baseline comparisons. Bacterial survival was measured as CFUs.
(0.13 MB TIF)

Figure S8 Live/dead staining of tobramycin-treated PA14 flow cell biofilms. 4 d old flow cell biofilms were treated with 1 µg/ml of tobramycin for 24 h. Treated biofilms were stained with Syto 9 (green) and propidium iodide (red) to visually assess live and dead cells. Images were taken from a 20× objective.
(2.14 MB TIF)

Figure S9 *pelA* expression is induced throughout biofilm growth. Biofilm cells are grown in a tube biofilm, with an initial attachment period of 30 min followed by continuous flow for 48 h. *pelA* transcripts are normalized to *ampR* transcript levels and then to the planktonic condition at time 0 h. Results shown are the mean of three independent experiments. Error bars represent the standard deviations.
(0.07 MB TIF)

Table S1 A list of primers, plasmids and bacterial strains used in the study.
(0.05 MB XLS)

Text S1 Supplementary text describing some of the materials and methods utilized.
(0.08 MB DOC)
