# Supplementary figures and images for: The Pel Polysaccharide Can Serve a Structural and Protective Role in the Biofilm Matrix of Pseudomonas aeruginosa
Source: PLoS Pathog. 2011 Jan 27;7(1):e1001264. doi: 10.1371/journal.ppat.1001264 (PMC3029257; doi:10.1371/journal.ppat.1001264)

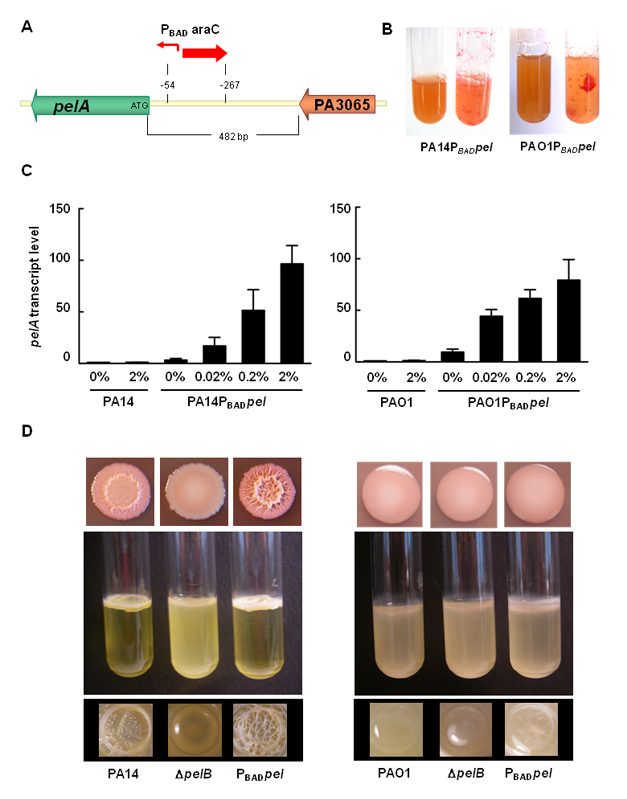

Supplement: Figure S1 — Generation of a pel-conditional strain. (A) Schematic drawing showing the replacement of the promoter region with an arabinose promoter on the chromosome to produce PAO1PBAD pel. (B) Overnight growth in the presence of 1% arabinose to PA14PBAD pel and PAO1PBAD pel leads to visual aggregates in liquid culture (right tubes) but not seen in the uninduced culture for either PA14PBAD pel and PAO1PBAD pel (left tubes). Congo red was added to visually enhance the phenotype. (C) Quantitative RT-PCR analysis of pelA transcription in PA14 and PA14PBAD pel (left) and PAO1 and PAO1PBAD pel(right) in the presence of increasing concentrations of arabinose. pelA transcription is normalized to ampR transcription. Results shown are the mean of three independent experiments. Error bars represent the standard deviations. (D) Colony morphology (top) and pellicle formation (middle, side-view; bottom, top-down) of PA14, PA14ΔpelB and PA14PBAD pel (left) and PAO1, PAO1ΔpelB and PAO1PBAD pel (right) grown in LB without NaCl containing 0.5% arabinose. Photographs were taken after five d of growth at room temperature. (0.42 MB TIF) [file ppat.1001264.s001.tif]

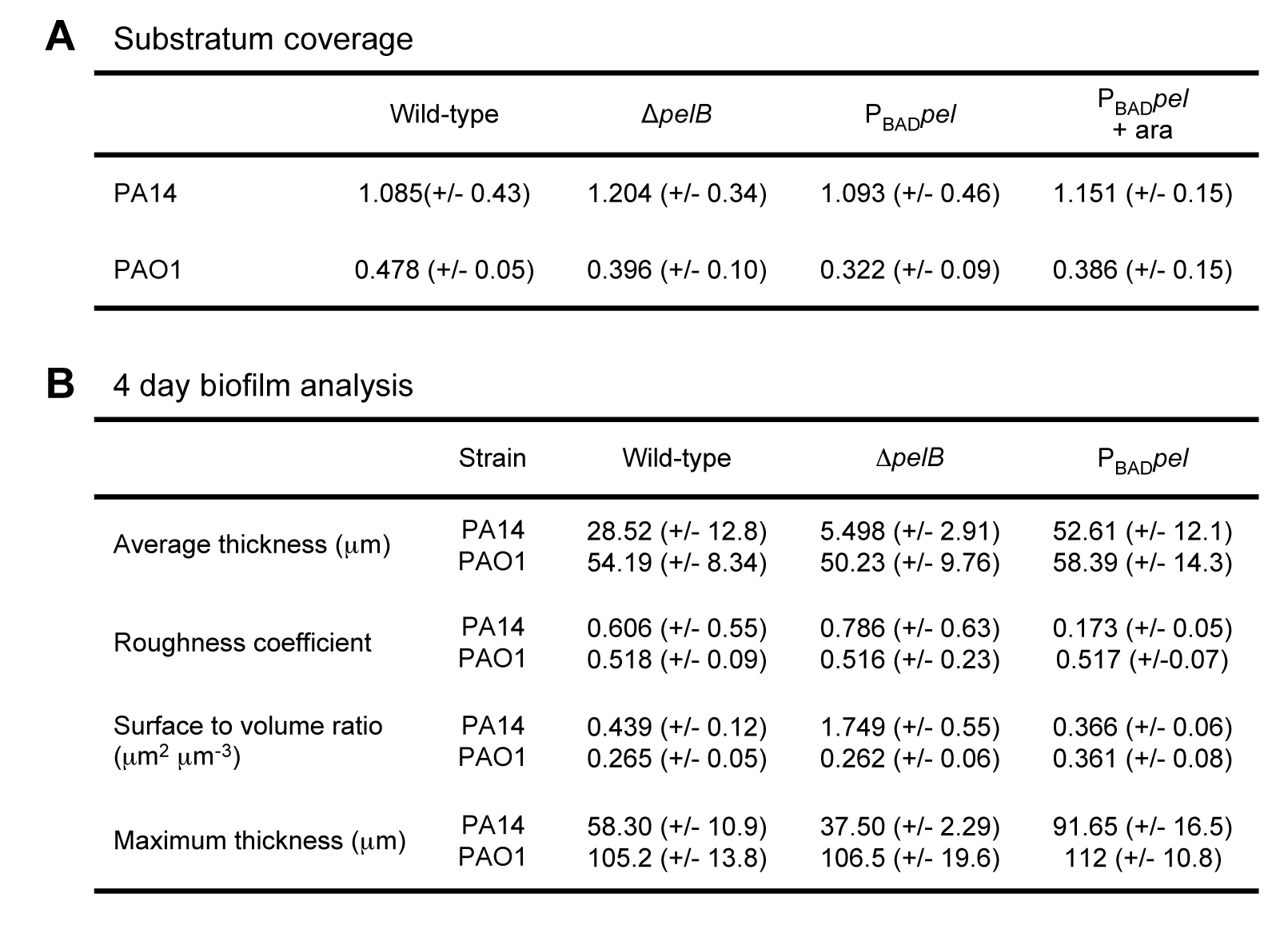

Supplement: Figure S2 — Attachment and biofilm structure quantified by COMSTAT 1. (A) Using the substratum coverage variable in COMSTAT 1, the relative number of cells attached to the glass slide of a flow cell after one hour of attachment followed by one hour of continuous flow was measured. Four images per flow cell done in duplicate in three independent experiments were evaluated. (B) COMSTAT 1 assessed four variables of biofilm structure for PA14, PA14ΔpelB, PA14PBAD pel, PAO1, PAO1ΔpelB and PAO1PBAD pel for the SCLM image stacks of day four biofilms. Three images per flow cell done in duplicate in three independent experiments were evaluated. (0.18 MB TIF) [file ppat.1001264.s002.tif]

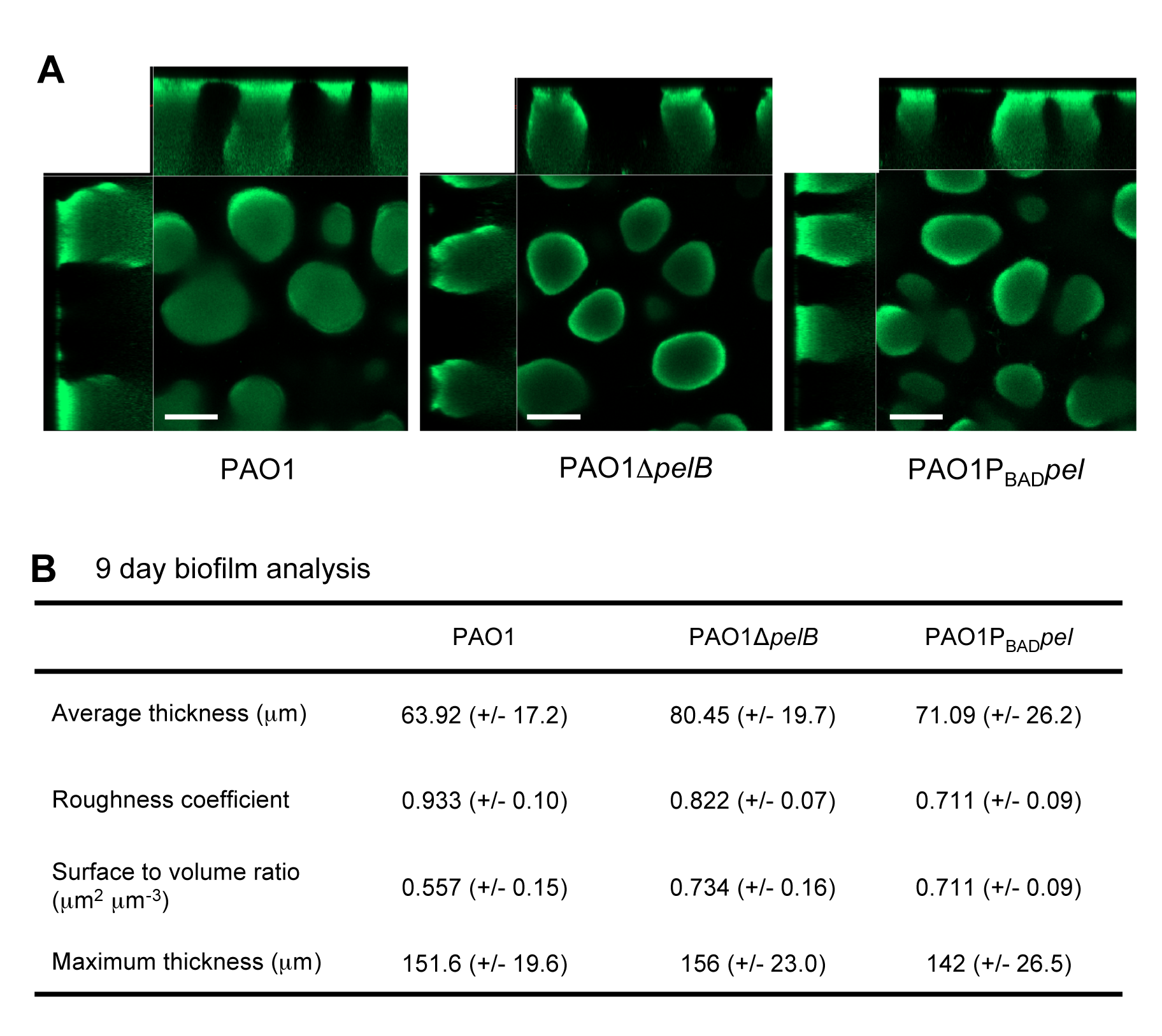

Supplement: Figure S3 — Biofilm structure in nine-day PAO1 biofilms. (A) Biofilm structure was visualized by SCLM in a flow cell after 9 days of growth. Representative top-down and side-view images are shown for PAO1, PAO1ΔpelB and PAO1PBADpel. Images were obtained using a 20× objective. Scale bars represent 100 µm. (B) Images were quantified for average thickness, roughness coefficient, surface to volume ratio and maximum thickness by COMSTAT 1. Four image stacks per flow cell done in duplicate in two independent experiments were evaluated. (0.97 MB TIF) [file ppat.1001264.s003.tif]

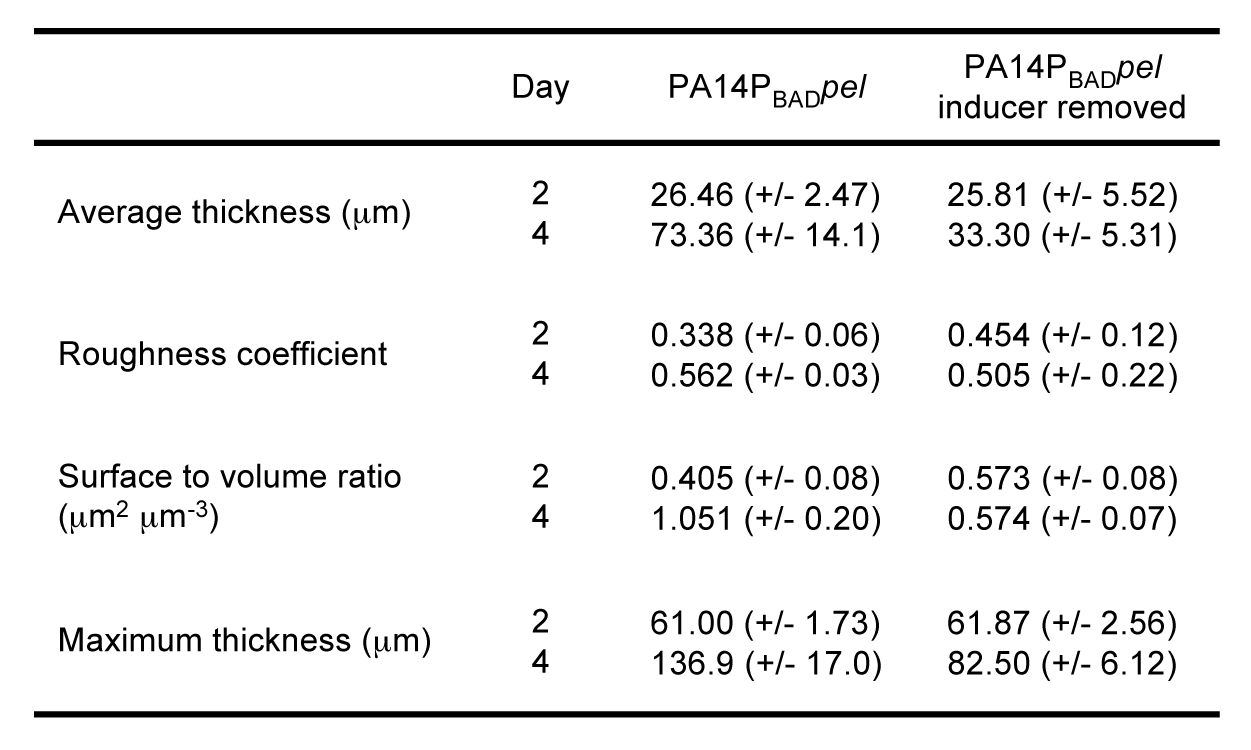

Supplement: Figure S4 — COMSTAT 1 analysis of arabinose removal in PA14PBAD pel flow cell biofilms. PA14PBAD pel biofilms were grown for two days under inducing conditions (0.2% arabinose). Biofilms were either continued to be grown in the presence or absence of the inducer, arabinose. COMSTAT 1 evaluated SCLM images for average thickness, roughness coefficient, surface to volume ratio and maximum thickness. Four image stacks per flow cell done in duplicate in two independent experiments were evaluated. (0.11 MB TIF) [file ppat.1001264.s004.tif]

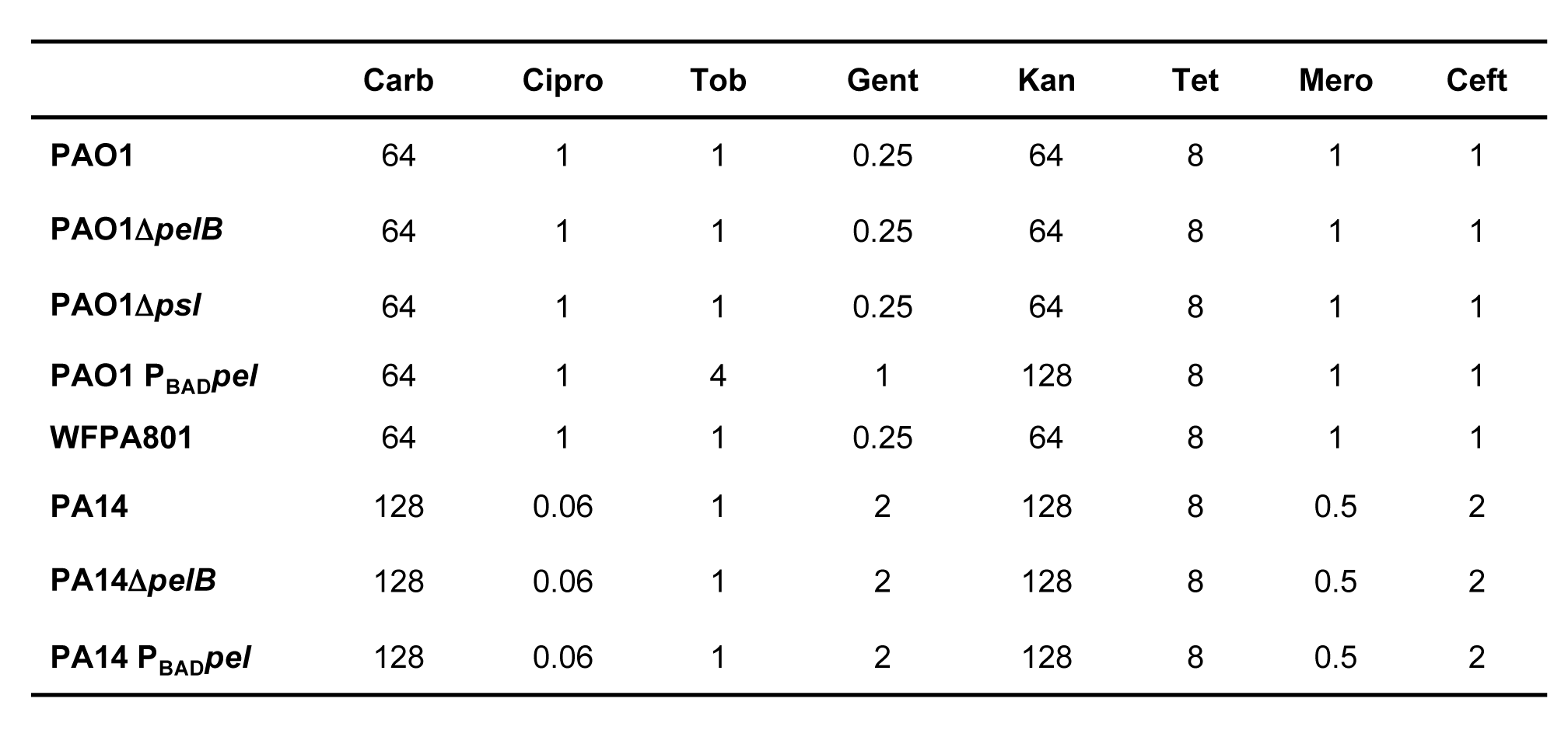

Supplement: Figure S5 — Effect of Pel on the minimum inhibitory concentration (MICs) to a wide range of antimicrobials. Strains were assessed for their MIC by broth dilution to carbenicillin (Carb), ciprofloxacin (Cip), tobramycin (Tob), gentamicin (Gent), tetracycline (Tet), meropenem (Mero), kanamycin (Kan) and ceftazidime (Ceft). Concentrations shown are in µg/ml and were empirically determined. Bacterial strains were grown in the presence of 0.5% arabinose. (0.11 MB TIF) [file ppat.1001264.s005.tif]

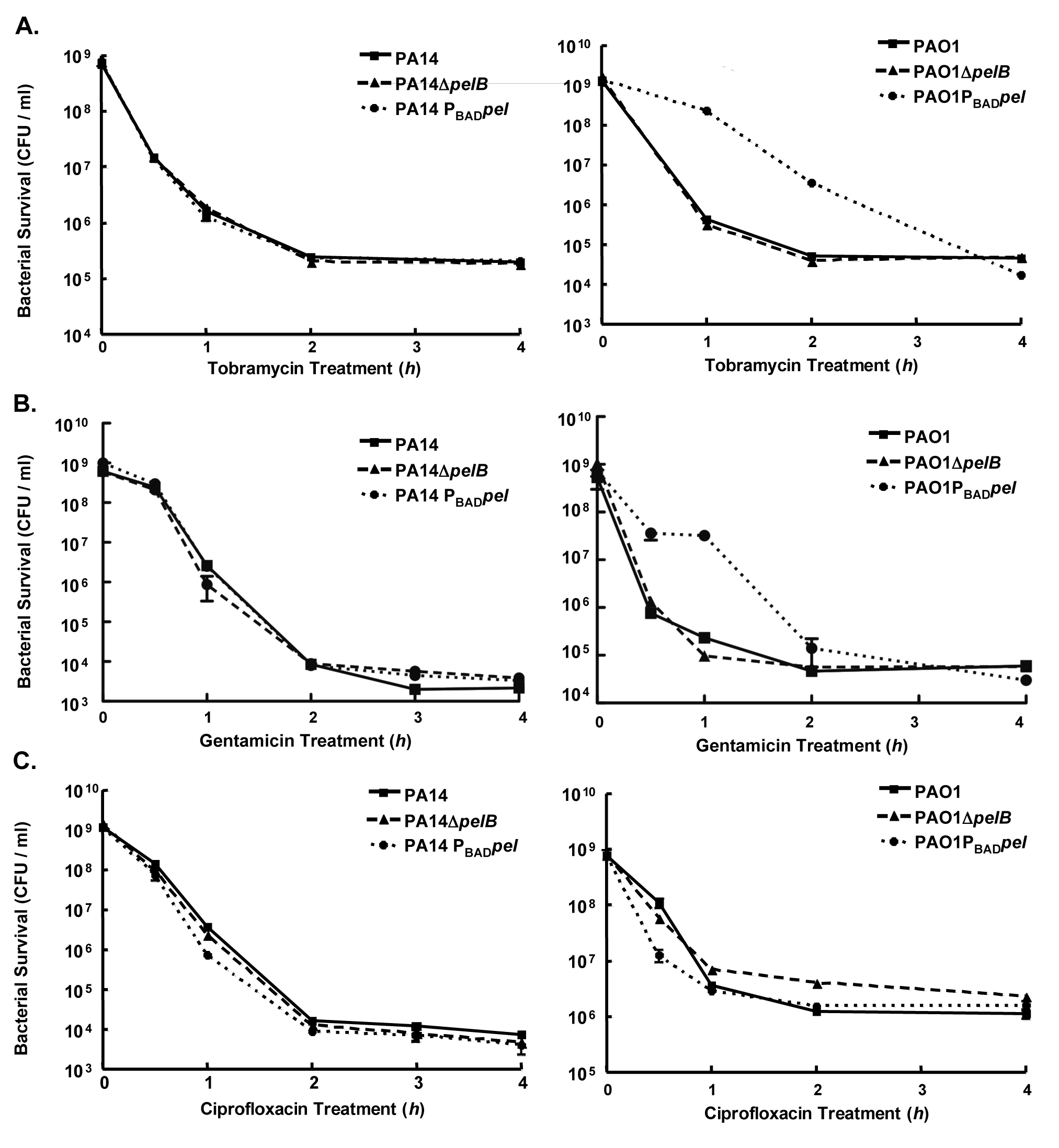

Supplement: Figure S6 — Analysis of Pel-mediated antibiotic tolerance in planktonic culture. Log-phase planktonic cultures were treated with either tobramycin (A), gentamicin (B) or ciprofloxacin (C). Bacterial survival was monitored over time by assessing the number of CFUs. On the left, PA14 (solid line), PA14ΔpelB (dashed line) and PA14PBAD pel (dotted line) were treated with 5 µg/ml tobramycin, 2 µg/ml gentamicin and 0.1 µg/ml ciprofloxacin. On the right, PAO1 (solid line), PAO1ΔpelB (dashed line) and PAO1PBAD pel (dotted line) were treated with 5 µg/ml tobramycin, 5 µg/ml gentamicin and 1 µg/ml ciprofloxacin. (0.33 MB TIF) [file ppat.1001264.s006.tif]

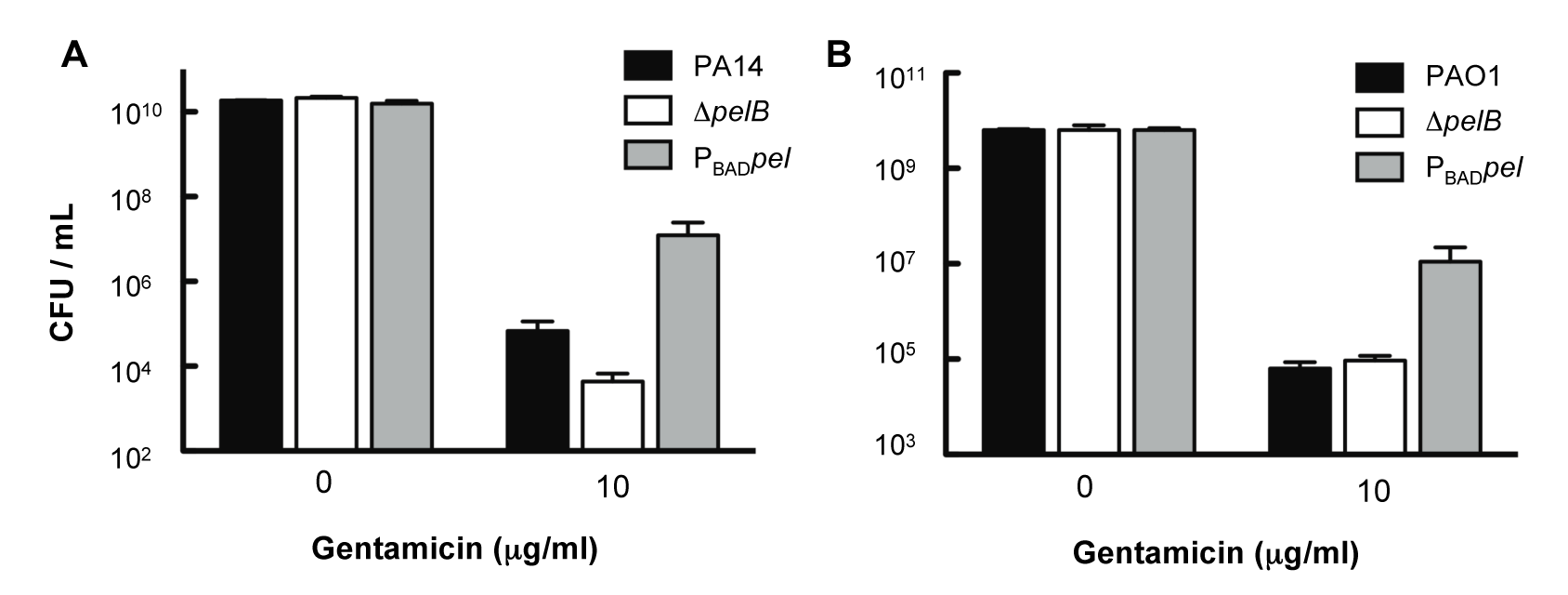

Supplement: Figure S7 — Pel provides tolerance to gentamicin during biofilm growth. 48 h colony biofilms for PA14, PA14ΔpelB and PA14PBADpel (A) and PAO1, PAO1ΔpelB and PAO1PBADpel (B) were assessed for antibiotic susceptibility. Biofilms were treated with gentamicin for 24 h. No antibiotic addition is included for baseline comparisons. Bacterial survival was measured as CFUs. (0.13 MB TIF) [file ppat.1001264.s007.tif]

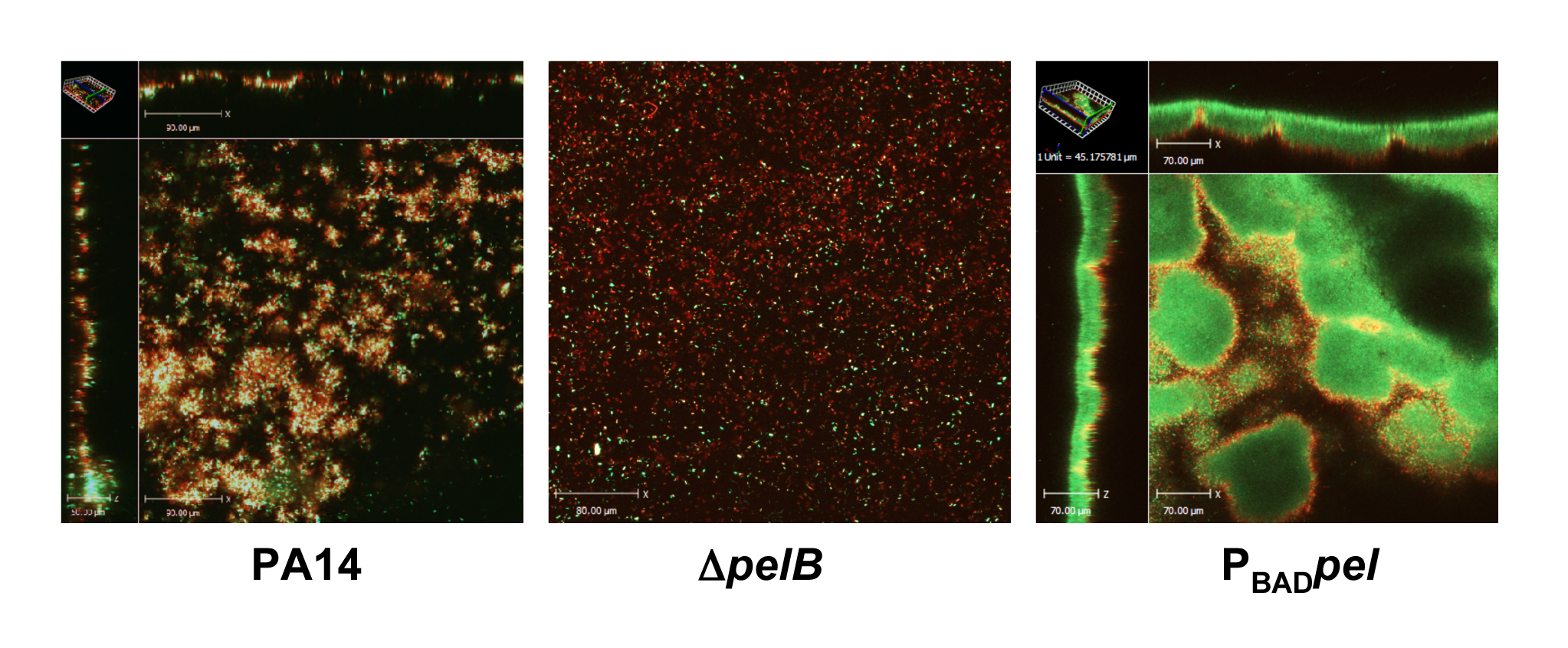

Supplement: Figure S8 — Live/dead staining of tobramycin-treated PA14 flow cell biofilms. 4 d old flow cell biofilms were treated with 1 µg/ml of tobramycin for 24 h. Treated biofilms were stained with Syto 9 (green) and propidium iodide (red) to visually assess live and dead cells. Images were taken from a 20× objective. (2.14 MB TIF) [file ppat.1001264.s008.tif]

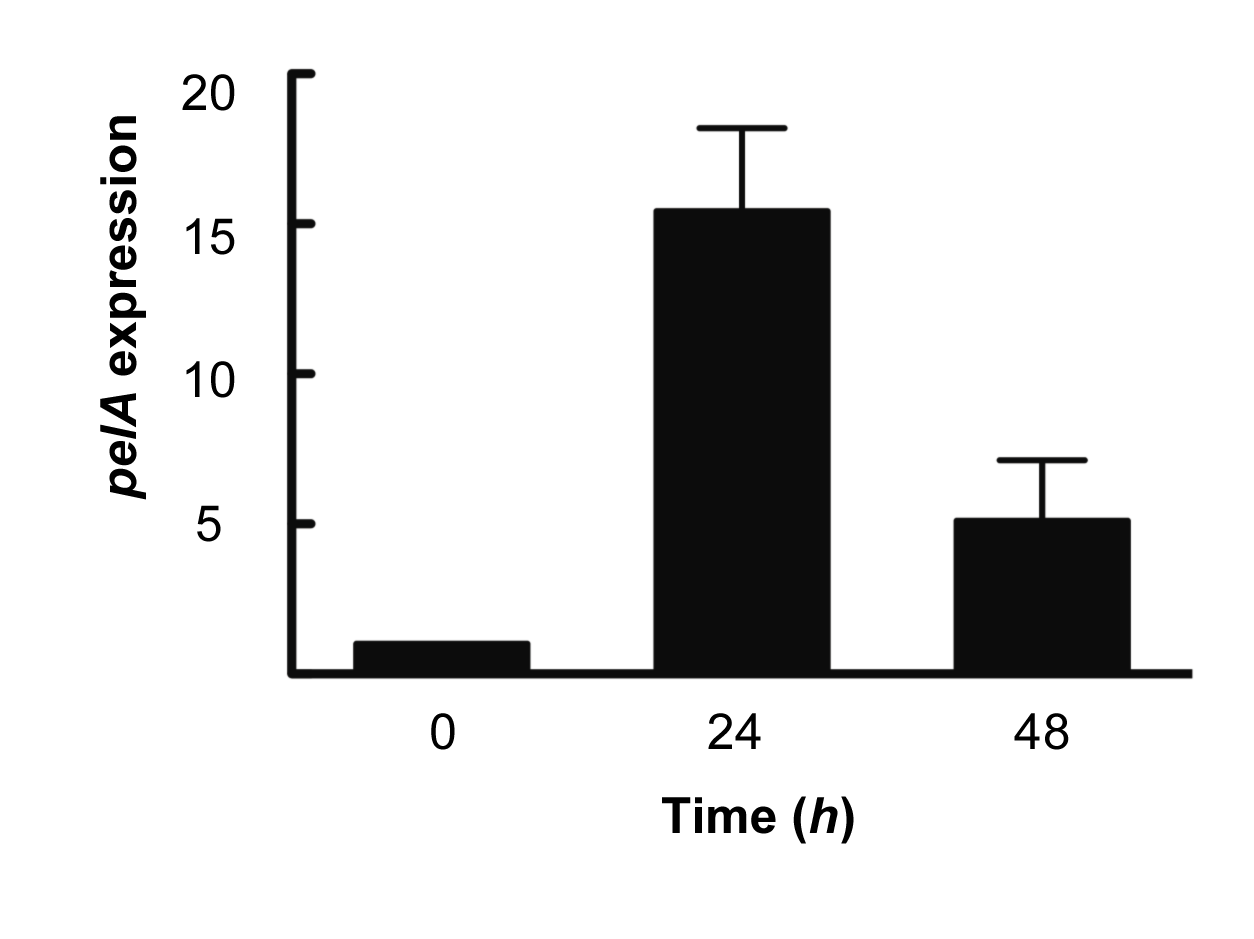

Supplement: Figure S9 — pelA expression is induced throughout biofilm growth. Biofilm cells are grown in a tube biofilm, with an initial attachment period of 30 min followed by continuous flow for 48 h. pelA transcripts are normalized to ampR transcript levels and then to the planktonic condition at time 0 h. Results shown are the mean of three independent experiments. Error bars represent the standard deviations. (0.07 MB TIF) [file ppat.1001264.s009.tif]
