# Supplementary material for: The Pel Polysaccharide Can Serve a Structural and Protective Role in the Biofilm Matrix of Pseudomonas aeruginosa
Source: PLoS Pathog. 2011 Jan 27;7(1):e1001264. doi: 10.1371/journal.ppat.1001264 (PMC3029257; doi:10.1371/journal.ppat.1001264)
Supplement: Text S1 — Supplementary text describing some of the materials and methods utilized. (0.08 MB DOC) [file ppat.1001264.s011.doc]

**SI Materials and Methods**

**Bacterial strains, strain construction, and growth media**

PAO1Δ*pelB* and PA14Δ*pelB* allelic replacement strains were constructed by using an unmarked, nonpolar deletion strategy described previously[1]. Flanking regions of *pelB* were amplified using primers *pelB* UP and *pelB* DN. The resultant PCR product was ligated into the suicide vector, pEX18gm, via HindIII restriction site. The plasmid pEX18gmΔ*pelB* was verified by sequencing analysis and mated into PAO1 and PA14. Single recombination mutants were selected on VBMM containing 100 µg/ml gentamicin. Double recombination mutants were selected on LB plates containing 5% sucrose and confirmed by PCR.

Construction of PAO1P_BAD_*pel* and PA14P_BAD_*pel* was constructed similarly to the previously described protocol [1]. In order to create a *pel*-inducible strain, we removed the promoter (positions -54 to -267 upstream of *pelA* for PAO1 and positions -72 to -249 for PA14) and replaced with an arabinose-inducible promoter, araC-P_BAD_ amplified from pJN105 [2]. Sewing PCR was used to construct a three-portion product including flanking regions of the native *pel* promoter and the araC-P_BAD_ promoter. The resultant PCR product was ligated into pEX18gm. Both completed pEX18gm-P_BAD_*pel* were verified by sequencing analysis and double recombinant mutants were generated as described above.

To complement a *pel* mutation, the entire *pel* operon was amplified using pel operon SpeI F and R primers. The resultant PCR product was ligated in the expression vector, pMJT-1, via SpeI restriction site [3]. The plasmid pMJT-1 P*pel* was verified by sequence analysis and transformed into PA14 and PA14Δ*pelB*.

PelF expression vector was constructed by amplifying full-length PelF with primers that omit the PelF stop codon and subsequently ligated into pET20b(+) using NdeI and XhoI restriction sites. This design removed the pelB leader sequence and constructed a PelF C-terminal 6x-HIS fusion. The construct was transformed into BL21 cells for purification.

**Flow Cell Bioreactor.** *P. aeruginosa* PA14 monocultures were tagged with GFP on the chromosome using the mini Tn7 system to allow fluorescent visualization of growing biofilms [4]. PAO1 monoclutures were tagged with GFP expression vector pMRP9-1 [5]. Log phase cultures grown in full-strength TSB were diluted to a final OD_600_ of 0.05 for PA14 and 0.01 for PAO1 in 1% TSB. Bacteria were inoculated into an inverted flow cell and allowed to attach for one hour. Upon initiation of media flow, fresh media of 1% TSB supplemented with 0.2% arabinose was pumped at a constant rate. Biofilms were grown at room temperature up to 9 d. Attachment images were taken one hour after flow was initiated. Cells were visualized by fluorescence by scanning confocal laser microscopy and image series were compiled using Velocity (Improvision) and analyzed using COMSTAT 1 software [6].

**Tube Biofilm.** Tube biofilms were conducted as previously described [7,8]. *P. aeruginosa* was grown to log phase in LB at 37°C shaking, diluted to an OD_600_ 0.05 and injected into a 6.35mm inner-diameter tube and incubated for 30 minutes. Control cells were incubated with static conditions in a 1.5ml tube. Biofilms were grown at room temperature in LB medium containing 100mM MOPS pH 7.0 at 50ml/h flow rate for 48 hours. The tube biofilms total RNA was isolated by opening the tube and scraping the adhered biomass using a cell scraper (Costar).

**PelF purification, antisera production and immunoblot analysis.** BL21 pET20b(+) PelF was grown to an OD_600_ 0.5 in LB supplemented with 100 µg/ml ampicillin at 37°C. The culture was induced with 0.5 mM IPTG overnight at 18°C. Cells were centrifuged and resuspended in 50mM Tris, pH 7.5, 500 mM NaCl and 10% glycerol. Cells were lysed by French press and protein was purified by nickel affinity chromatography and size exclusion chromatography using an FPLC. Fractions with purified PelF were combined and concentrated to 1 mg/ml. Antisera was generated in rabbits using a 70-day standard protocol (Open Biosystems). Antisera was absorbed using PAO1Δ*pelF* lysates based on standard protocols [9]. Immunoblots were performed with whole-cell lysates as described with equal amounts of total protein in each lane [9]. Protein concentration was measured using the Pierce 660 nm protein assay (Thermo Scientific). 10 μg of total protein was loaded onto a 7.5% pre-cast SDS-PAGE gel (Bio-rad). Proteins were transferred to a PVDF membrane. Membranes were blocked using 5% non-fat milk in Tris buffered saline containing 0.1% Tween (TBST) and probed with absorbed rabbit polyclonal α-PelF antibody (1:2,500). Blots were washed three times with TBST, probed with α-rabbit horseradish peroxidase-conjugated antibodies (1:25,000, Thermo Scientific) and washed three times with TBST. Membranes were incubated with the Pierce SuperSignal West Pico ECL reagent (Thermo Scientific) and detected using X-ray film (Kodak).

**References:**

1. Hoang TT, Karkhoff-Schweizer RR, Kutchma AJ, Schweizer HP (1998) A broad-host-range Flp-FRT recombination system for site-specific excision of chromosomally-located DNA sequences: application for isolation of unmarked Pseudomonas aeruginosa mutants. Gene 212: 77-86.

2. Newman JR, Fuqua C (1999) Broad-host-range expression vectors that carry the L-arabinose-inducible *Escherichia coli* araBAD promoter and the araC regulator. Gene 227: 197-203.

3. Kaneko Y, Thoendel M, Olakanmi O, Britigan BE, Singh PK (2007) The transition metal gallium disrupts *Pseudomonas aeruginosa* iron metabolism and has antimicrobial and antibiofilm activity. J Clin Invest 117: 877-888.

4. Koch B, Jensen LE, Nybroe O (2001) A panel of Tn7-based vectors for insertion of the gfp marker gene or for delivery of cloned DNA into Gram-negative bacteria at a neutral chromosomal site. J Microbiol Methods 45: 187-195.

5. Davies DG, Parsek MR, Pearson JP, Iglewski BH, Costerton JW, et al. (1998) The involvement of cell-to-cell signals in the development of a bacterial biofilm. Science 280: 295-298.

6. Heydorn A, Ersboll BK, Hentzer M, Parsek MR, Givskov M, et al. (2000) Experimental reproducibility in flow-chamber biofilms. Microbiology 146 ( Pt 10): 2409-2415.

7. Kirisits MJ, Prost L, Starkey M, Parsek MR (2005) Characterization of colony morphology variants isolated from *Pseudomonas aeruginosa* biofilms. Appl Environ Microbiol 71: 4809-4821.

8. Schaefer AL, Greenberg EP, Parsek MR (2001) Acylated homoserine lactone detection in *Pseudomonas aeruginosa* biofilms by radiolabel assay. Methods Enzymol 336: 41-47.

9. Hermodson M (1996) Current protocols in protein science, edited by J.E. Coligan, B.M. Dunn, H.L. Ploegh, D.W. Speicher, and P.T. Wingfield. New York: Wiley, 1995, 864 pages (core) + 576 pages (4 supplements)/year (updated looseleaf), print and CD-ROM. Proteins: Structure, Function, and Bioinformatics 24: 409-409.
